# Comparative efficacy and safety of Chinese herbal injections combined with the FOLFOX regimen for treating gastric cancer in China: a network meta-analysis

**DOI:** 10.18632/oncotarget.20320

**Published:** 2017-08-18

**Authors:** Dan Zhang, Jiewen Zheng, Mengwei Ni, Jiarui Wu, Kuaihuan Wang, Xiaojiao Duan, Xiaomeng Zhang, Bing Zhang

**Affiliations:** ^1^ Department of Clinical Chinese Pharmacy, School of Chinese Materia Medica, Beijing University of Chinese Medicine, Beijing 100102, China

**Keywords:** Chinese herbal injections, FOLFOX regimen, gastric cancer, network meta-analysis

## Abstract

**Background:**

Chinese herbal injections (CHIs) have been proven beneficial to patients with gastric cancer for improving clinical efficacy and relieving adverse reactions (ADRs) of chemotherapy. A network meta-analysis (NMA) was conducted in this study to assess the comparative efficacy and safety of CHIs combined with FOLFOX regimen for treating gastric cancer.

**Results:**

A total of 2316 records were searched, and 81 eligible RCTs involving 15 types of CHIs and 5978 patients were included in the NMA. The results showed that patients who received Shengqifuzheng+ FOLFOX, Compound kushen+ FOLFOX, Huachansu+ FOLFOX, Astragalus+ FOLFOX, Kangai+ FOLFOX, and Lentinan injection + FOLFOX could significantly improve clinical efficacy than using FOLFOX single, and their odds ratios (OR) and 95% confidence intervals (CI)s were 1.57 (1.19,2.09), 2.12 (1.62,2.78),1.72 (1.08,2.80), 3.06 (1.01,8.99), 2.01 (1.52,2.70), and 1.99 (1.20,3.38) respectively. Furthermore, the therapy of Aidi+ FOLFOX, Shenqifuzheng+ FOLFOX, Compound Kushen+ FOLFOX, Huachansu+ FOLFOX, Astragalus polysaccharides+ FOLFOX, Kangai+ FOLFOX, Ginseng polysaccharide+ FOLFOX, Lentinan+ FOLFOX, Xiaoaiping+ FOLFOX, and Shenmai injection + FOLFOX could also achieve a higher performance status compared with FOLFOX regimen alone. Similarly, patients who received CHIs combine with FOLFOX regimen were associated with a significantly decrease the incidence of leucopenia, gastrointestinal reaction and hepatic dysfunction. Cluster analysis demonstrated that Astragalus polysaccharides+ FOLFOX, and Kangai+ FOLFOX seemed optimal therapies in improving clinical efficacy and performance status; Astragalus polysaccharides+ FOLFOX was superior in reducing leucopenia and gastrointestinal reaction; Disodium Cantharidinate and Vitamin B6+ FOLFOX was associated with favorable effects in reducing gastrointestinal reaction and hepatic dysfunction. By contrary, receiving FOLFOX regimen single was proved to rank the worst for these outcomes.

**Materials and Methods:**

A comprehensive literature search was performed in several electronic databases to identify randomized controlled trial (RCTs) regarding CHIs for gastric cancer until January 10, 2017. The quality assessment was accomplished according to the Cochrane risk of bias tool and the methodological section of the CONSORT statement. And a random-effects model NMA was utilized to compare different CHIs combined with FOLFOX regimen with regard to efficacy and safety. Data were analyzed using STATA 12.0 and Win-BUGS 1.4 software.

**Conclusions:**

The results of this NMA suggested that among 15 types of CHIs, Astragalus polysaccharides injection combined with FOLFOX regimen seemed optimal for patients with gastric cancer in improving clinical efficacy and performance status, and relieving ADRs. However, our findings should be confirmed by more prospectively designed, large-sample and multi-center RCTs.

## INTRODUCTION

Gastric cancer is a leading cause of cancer-related deaths worldwide; its morbidity is in the first place of gastrointestinal malignancies [1–3]. Currently, gastric cancer has the characteristics of high morbidity and mortality, low early diagnosis rate, surgical resection rate and 5 years survival rate [4–6]. The etiology of gastric cancer has not yet been elucidated, whereas it is reported that the development of gastric cancer maybe related with chemical carcinogen, micronutrient deficiency, microbial infection, hereditary and other factors [7]. Surgical treatment is the therapeutic modality that offers the greatest possibility of cure for patients with gastric cancer, besides, chemotherapy and radiotherapy are important therapeutic options for patients who are suffering from distant metastases or unable to receive surgery [8–9]. FOLFOX regimen is one of the internationally recognized first-line chemotherapy regimen for gastric cancer, and this regimen is composed of 5-fluorouracil (5-Fu), leucovorin (LV) and oxaliplatin (L-OHP) [7, 10]. According to the relevant studies, L-OHP and 5-Fu may cause ADRs such as peripheral neurotoxicity, pancytopenia and severe gastrointestinal toxicity [11, 12]. It has been thousands of years that traditional Chinese medicine (TCM) are applied at clinical to treating cancers in China, Japan, and other Asian countries [13–14]. As complementary and alternative medicine, TCM has become one of main methods for cancer comprehensive treatment, because it has the advantages of improving the clinical symptoms, enhancing immunity and alleviating chemotherapy-induced ADRs [15, 16]. Meanwhile, TCM could have therapeutic effects in toxicity reducing and efficacy enhancing when it is in combination with radiotherapy or chemotherapy [17]. The results of a network pharmacological study present that 13 Chinese herbs were associated with survival benefit for patients with stage IV gastric cancer through correlation analysis [18]. Based on an orthotopic mouse model of human gastric cancer, the pharmacological study confirms that ginsenoside Rg3 could and reduce lymphatic metastasis and inhibit tumor growth, those may be related to suppress expression of vascular endothelial growth factor receptor-C, lymphogeneous metastasis and vascular endothelial growth factor-D [19]. Another pharmacological study reveals that the compounds which isolated from *Sophora Flavescens* could induce mitochondria-mediated apoptosis in human gastric cancer cells, the possible mechanisms might be the induction of cell cycle arrest and apoptosis [20]. And through the nationwide survey in China, the study indicates that 42.4% of 51,382 cancer patients use 33 anticancer Chinese patent medicines and 24.8% of the cancer patients used both anticancer Chinese patent medicines and anticancer western medicines [21]. Furthermore, CHIs possess the advantages of high bioavailability, high curative effects compared with TCM decoction [22].

Although NMA is developed from the standard meta-analysis, it allows for the simultaneous evaluation of multiple interventions to provide more comprehensive and valuable information for clinical decision-making through both direct and indirect comparisons [23–25]. Moreover, NMA could sort the different interventions based on their therapeutic effects and the probability of optimal interventions [24–25]. Given the lack of head-to-head RCTs between different CHIs, a NMA was conducted to compare the efficacy and safety of multiple CHIs combined with FOLFOX regimen simultaneously to investigate which is the best CHI for gastric cancer.

## RESULTS

### Literature search and baseline characteristics

**Figure 1:**
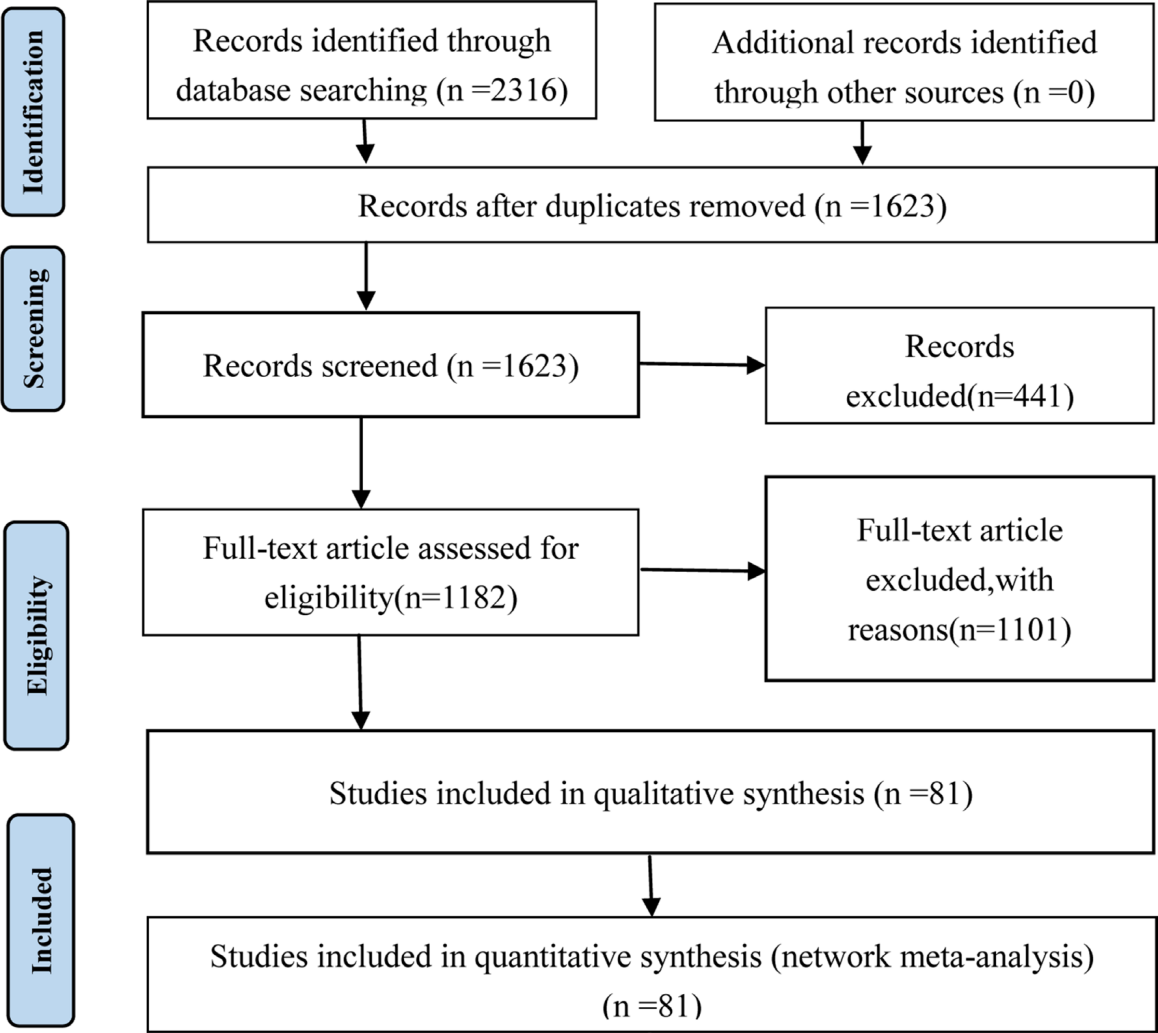
PRISMA flow diagram

As illustrated by the PRISMA flow diagram (Figure 1), a total of 2316 suitable articles were identified via a primary search of the aforementioned literature databases. According to inclusion and exclusion criteria, the appropriate trails were selected strictly. Finally, 81 eligible RCTs involving 15 types of CHIs and 5978 patients were included in the NMA, and the included RCTs were all published in Chinese [26–106]. 15 types of CHIs were identified, including Astragalus injection (1 trial), Shenmai injection (1 trial), Disodium cantharidinate and vitamin B6 injection (1 trial), Delisheng injection (1 trial), Elemene injection (1 trial), Placental peptide injection (1 trial), Xiaoaiping injection (2 trial), Ginseng polysaccharide injection (2 trial), Astragalus polysaccharide injection (2 triasl), Lentinan injection (5 trials), Hauchansu injection (6 trials), Aidi injection (9 trials), Kangai injection (14 trials), Compound kushen injection (17 trials), Shenqifuzheng injection (18 trials).

**Figure 2:**
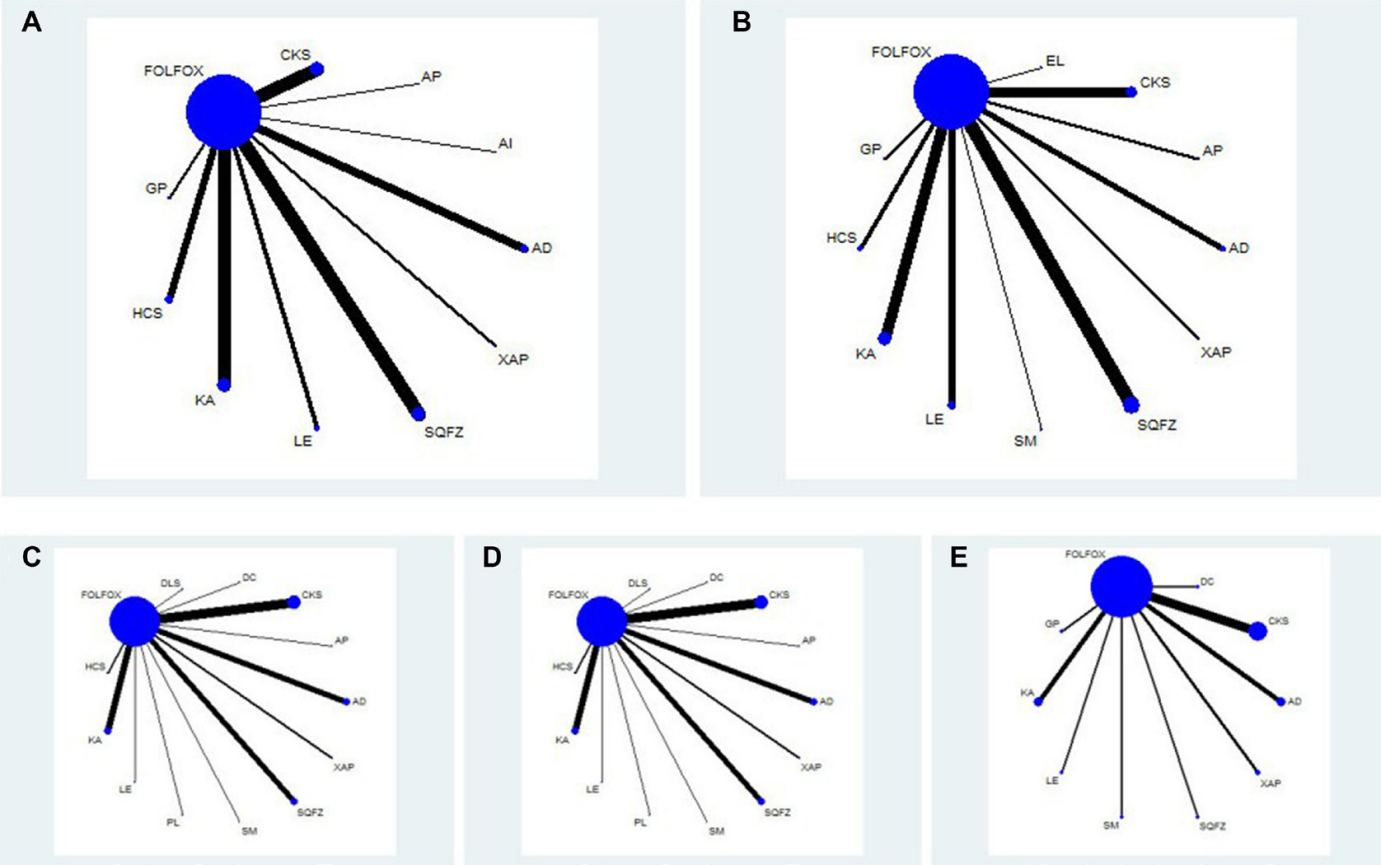
Network graph for 5 outcomes Note: (**A**) Clinical efficacy; (**B**) Performance status; (**C**) Leucopenia; (**D**) Gastrointestinal reaction; (**E**) Hepatic dysfunction; FOLFOX: FOLFOX Chemotherapy regimen; CKS: Compound kushen injection; AP: Astragalus polysaccharide injection; AI: Astragalus injection; AD: Aidi injection; XAP: Xiaoaiping injection; SQFZ: Shenqifuzheng injection; LE: Lentinan injection; KA: Kangai injection; HCS: Huachansu injection; GP: Ginseng Polysacchride Injection; EL: Elemene injection; SM: Shenmai injection; DLS: Delisheng injection; DC: Disodium cantharidinate and vitamin B6 injection; PL: Placenta polypeptide injection.

The 81 RCTs included 15 types of CHIs and 5978 patients with gastric cancer, among 3049 patients were in CHIs group and 2929 patients were in FOLFOX groups. All of the included RCTs reported patient numbers and ages. Moreover, 74 (91.36%), 52 (64.20%), 49 (60.49%) and 56 (69.14%) trials respectively described the patients’ gender, tumor staging, expected survival time and Karnofsky performance score (KPS) before treatment. More details of baseline characteristics for individual trials were presented in Supplementary Table 1. Besides, the network graph revealing the distribution of RCTs and relationship of CHIs for 5 outcomes was depicted in Figure 2.

### Results of quality assessment

The results of quality assessment for all included RCTs were showed in Supplementary Table 2. Although all of the included RCTs mentioned randomization and described the inclusion and exclusion criteria, the calculation of sample size was not mentioned in each RCT. In terms of random sequence generation methods, 11 RCTs (13.58%) used a random number table, random lottery form was adopted to divide into groups in 1 trail (1.23%), 1 RCT (1.23%) adopted the method of odd-even number, and 2 RCTs (2.47%) applied the method of hospitalized time difference. Moreover, there were only 3 RCTs mentioning blinding method. In addition, 6 RCTs (7.41%), 8 RCTs (9.88%), 63 RCTs (77.78%) and 21RCTs (25.93%) provided the details about funding supports, follow-up, ADRs, and medical ethics.

### Outcomes

### The clinical efficacy

**Figure 3:**
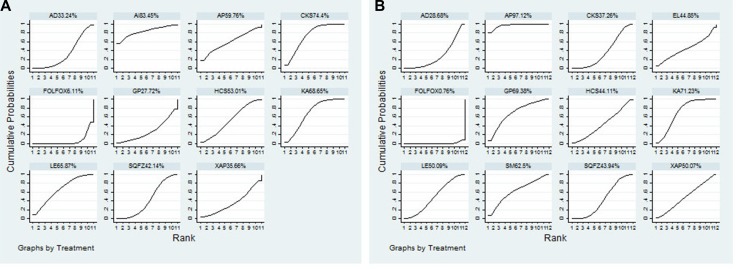
Rank of cumulative probabilities of clinical efficacy and performance status Note: (**A**) Clinical efficacy; (**B**) Performance status; FOLFOX: FOLFOX Chemotherapy regimen; AD: Aidi injection; AI: Astragalus injection; AP: Astragalus polysaccharide injection; CKS: Compound kushen injection; GP: Ginseng Polysacchride Injection; HCS: Huachansu injection; KA: Kangai injection; LE: Lentinan injection; SQFZ: Shenqifuzheng injection; XAP: Xiaoaiping injection.EL: Elemene injection; SM: Shenmai injection.

The data of clinical efficacy were available for 61 RCTs involving 10 CHIs. The results based on NMA demonstrated that patients who received Shengqifuzheng+ FOLFOX (OR = 1.57, 95% CI = 1.19–2.09), Compound kushen+ FOLFOX (OR = 2.12, 95% CI = 1.62–2.78), Huachansu+ FOLFOX (OR = 1.72, 95% CI = 1.08–2.80), Astragalus+ FOLFOX (OR = 3.06, 95% CI = 1.01–8.99), Kangai+ FOLFOX (OR = 2.01, 95% CI = 1.52–2.70), and Lentinan injection + FOLFOX (OR = 1.99, 95% CI = 1.20–3.38) could significantly improve clinical efficacy than using FOLFOX single. Besides, on the basis of combining with FOLFOX regimen, there was without significantly difference between different CHIs. The OR along with 95% CI of each intervention for clinical efficacy was presented in Table 1. According to the calculated probabilities for clinical efficacy, the 10 types of CHIs were ranked as follows: Astragalus (83.45%, 1 trail) > Compound kushen (74.4%, 13 trails) > Kangai (68.65%, 12 trails) > Lentinan (65.87%, 4 trails) > Astragalus polysaccharides (59.76%, 1 trails)> Huachansu (53.01%, 6 trails) > Shenqifuzheng (42.14%, 13 trails) > Xiaoaiping (35.66%, 2 trails) > Aidi (33.24%, 7 trails) > Ginseng polysaccharide (27.72%, 2 trails). The rank of cumulative probabilities for clinical efficacy was revealed in Figure 3A.

**Table 1:** Network meta-analysis results of clinical efficacy (upper right quarter) and performance status (lower left quarter)

**AD + FOLFOX**	0.70 (0.49,1.01)	1.11 (0.69,1.75)	1.50 (0.94,2.32)	1.23 (0.67,2.18)	1.35 (0.49,3.95)	2.16 (0.71,6.68)	1.42 (0.88,2.25)	0.89 (0.40,2.03)	1.41 (0.74,2.69)	0.98 (0.44,2.26)	--	--
**0.42 (0.24,0.75)**	**FOLFOX**	**1.57 (1.19,2.09)**	**2.12 (1.62,2.78)**	**1.72 (1.08,2.80)**	1.92 (0.72,5.24)	**3.057 (1.01,8.99)**	**2.01 (1.52,2.70)**	1.26 (0.61,2.62)	**1.99 (1.20,3.38)**	1.40 (0.68,2.87)	--	--
1.27 (0.63,2.51)	**2.99 (2.06,4.34)**	**SQFZ** **+ FOLFOX**	1.35 (0.90,2.00)	1.11 (0.63,1.95)	1.23 (0.44,3.47)	1.97 (0.63,5.96)	1.28 (0.84,1.91)	0.80 (0.37,1.76)	1.28 (0.72,2.34)	0.89 (0.41,1.95)	--	--
1.16 (0.56,2.38)	**2.74 (1.77,4.22)**	0.92 (0.51,1.63)	**CKS** **+ FOLFOX**	0.81 (0.47,1.44)	0.91 (0.33,2.60)	1.44 (0.46,4.44)	0.94 (0.64,1.42)	0.59 (0.27,1.32)	0.94 (0.53,1.73)	0.66 (0.31,1.43)	--	--
1.24 (0.46,3.43)	**2.94 (1.32,6.77)**	0.98 (0.41,2.48)	1.072 (0.43,2.77)	HCS + FOLFOX	1.11 (0.36,3.38)	1.77 (0.53,5.87)	1.16 (0.66,2.00)	0.73 (0.31,1.70)	1.15 (0.58,2.38)	0.80 (0.34,1.91)	--	--
**4.81 (1.64,14.91)**	**11.38 (4.62,30.17)**	**3.82 (1.44,10.85)**	**4.16 (1.53,12.08)**	**3.89 (1.13,13.6)**	**AP** **+ FOLFOX**	1.55 (0.35,7.05)	1.037 (0.37,2.81)	0.66 (0.19,2.42)	1.037 (0.34,3.10)	0.72 (0.21,2.44)	--	--
—	—	—	—	—	—	**AI** **+ FOLFOX**	0.66 (0.22,2.02)	0.42 (0.11,1.45)	0.65 (0.20,2.24)	0.46 (0.12,1.71)	--	--
1.82 (0.90,3.72)	**4.30 (2.91,6.50)**	1.44 (0.84,2.51)	1.57 (0.88,2.87)	1.46 (0.58,3.61)	0.38 (0.13,1.02)	—	**KA** **+ FOLFOX**	0.63 (0.29,1.39)	0.99 (0.56,1.83)	0.70 (0.32,1.51)	--	--
1.93 (0.66,5.78)	**4.55 (1.87,11.71)**	1.52 (0.58,4.23)	1.66 (0.62,4.70)	1.56 (0.45,5.33)	0.40 (0.11,1.49)	—	1.06 (0.39,2.92)	**GP** **+FOLFOX**	1.59 (0.63,3.97)	1.10 (0.39,3.15)	--	--
1.36 (0.61,3.09)	**3.23 (1.88,5.66)**	1.082 (0.56,2.12)	1.18 (0.59,2.42)	1.10 (0.40,2.94)	**0.28 (0.094,0.82)**	—	0.75 (0.38,1.48)	0.71 (0.24,2.05)	**LE** **+ FOLFOX**	0.70 (0.29,1.74)	--	--
1.37 (0.48,3.92)	**3.23 (1.36,7.85)**	1.085 (0.42,2.84)	1.18 (0.45,3.17)	1.096 (0.33,3.64)	0.28 (0.079,1.01)	—	0.75 (0.29,1.96)	0.71 (0.20,2.51)	1.00 (0.36,2.82)	**XAP** **+ FOLFOX**	--	--
1.74 (0.50,6.39)	**4.11 (1.34,13.33)**	0.73 (0.21,2.35)	0.67 (0.19,2.19)	0.72 (0.17,2.90)	2.77 (0.63,12.16)	—	1.05 (0.31,3.47)	1.11 (0.25,4.76)	0.78 (0.22,2.75)	0.79 (0.18,3.29)	**SM** **+ FOLFOX**	--
1.23 (0.28,5.94)	2.90 (0.73,12.68)	0.97 (0.23,4.45)	1.057 (0.25,4.91)	0.98 (0.20,5.19)	0.25 (0.048,1.42)	—	0.67 (0.16,3.10)	1.57 (0.29,8.19)	1.12 (0.23,4.91)	1.12 (0.20,5.78)	0.71 (0.12,4.48)	**EL +FOLFOX**

### Performance status

A total of 45 RCTs with 11 CHIs contributed to performance status analysis. The results indicated that the therapies of Aidi +FOLFOX (OR = 0.42, 95% CI = 0.24–0.75), Shenqifuzheng +FOLFOX (OR = 2.99, 95% CI = 2.06–4.34), Compound Kushen +FOLFOX (OR = 2.74, 95% CI = 1.77–4.22), Huachansu +FOLFOX (OR = 2.94, 95% CI = 1.32–6.77), Astragalus polysaccharides +FOLFOX (OR = 11.38, 95% CI = 4.62–30.17), Kangai +FOLFOX (OR = 4.30, 95% CI = 2.91–6.50), Ginseng polysaccharide +FOLFOX (OR = 4.55, 95% CI = 1.87–11.71), Lentinan +FOLFOX (OR = 3.23, 95% CI = 1.88–5.66), Xiaoaiping +FOLFOX (OR = 3.23, 95% CI = 1.36–7.85), Shenmai +FOLFOX (OR = 4.11, 95% CI = 1.34–13.33) were associated with favorable responses in terms of performance status compared with using FOLFOX regimen single, and significant difference was detected between these groups. Moreover, on the basis of combining with FOLFOX regimen, Astragalus polysaccharides was proven to significantly improve performance status than Aidi (OR = 4.81, 95% CI = 1.64–14.91), Shenqifuzheng (OR = 3.82, 95% CI = 1.44–10.85), Compound kushen (OR = 4.16, 95% CI = 1.53–12.08), Huachansu (OR = 3.89, 95% CI = 1.13–13.6), Lentinan (OR = 0.28, 95% CI = 0.094–0.82), and the difference between these CHIs groups was statistically significant (Table 1). As the Figure 3B displayed, the rankings of 11 CHIs based on their SUCRA value were: Astragalus polysaccharides (97.12%, 2 trails) > Kangai (71.23%, 8 trails) > Ginseng polysaccharide (69.38%, 2 trails) > Shenmai (62.5%, 1 trail) > Lentinan (50.09%, 5 trails) > Xiaoaiping (50.07%, 2 trails) > Elemene (44.85%, 1 trail) > Huachansu (44.11%, 3 trails) > Shenqifuzheng (43.94%, 10 trails) > Compound kushen (37.26%, 7 trails) > Aidi (28.68%, 4 trails).

### ADRs

### Leucopenia

**Figure 4:**
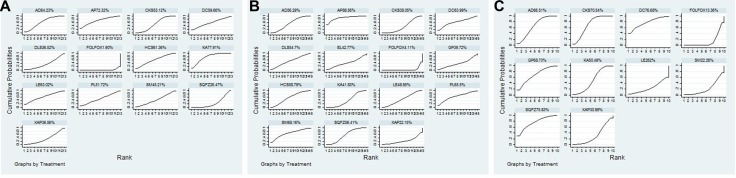
Rank of cumulative probabilities of ADRs Note: (**A**) Leucopenia; (**B**) Gastrointestinal reaction; (**C**) Hepatic dysfunction; FOLFOX:FOLFOX Chemotherapy regimen; AD: Aidi injection; AP: Astragalus polysaccharide injection; CKS: Compound kushen injection; DC: Disodium cantharidinate and vitamin B6 injection; DLS: Delisheng injection; HCS: Huachansu injection; KA: Kangai injection; LE: Lentinan injection; PL: Placenta polypeptide injection; SM: Shenmai injection; SQFZ: Shenqifuzheng injection; XAP: Xiaoaiping injection; EL: Elemene injection; GP: Ginseng Polysacchride Injection.

A total of 35 RCTs involving 12 CHIs provided data for leucopenia, the results revealed that: the therapies of Aidi +FOLFOX (OR = 3.64, 95% CI = 2.03–6.65), Shenqifuzheng +FOLFOX (OR = 0.51, 95% CI = 0.28–0.88), Compound kushen +FOLFOX (OR = 0.33, 95% CI = 0.21–0.49), Huachansu +FOLFOX (OR = 0.28, 95% CI = 0.099–0.77), Astragalus polysaccharides +FOLFOX (OR = 0.22, 95% CI = 0.060–0.74), Kangai +FOLFOX (OR = 0.22, 95% CI = 0.12–0.37), Lentinan +FOLFOX (OR = 0.26, 95% CI = 0.069–0.94) clearly stood better with regard to relieve leucopenia than only receiving FOLFOX regimen, with significant differences between these groups. Among CHIs groups, Kangai +FOLFOX yielded a better result for reducing leucopenia compared with Shenqifuzheng +FOLFOX (OR = 0.43, 95% CI = 0.19–0.94), and there was significant difference between two groups. The results of each intervention for leucopenia were provided in Table 2. According to cumulative probabilities for leucopenia in Figure 4A, 12 types of CHIs were ranked as follows: Kangai (77.91%, 6 trails) > Astragalus polysaccharides (72.32%, 1 trails) > Aidi (64.23%, 5 trails) > Lentinan (63.02%, 1 trails) > Hauchansu (61.36%, 2 trails) > Disodium cantharidinate and vitamin B6 (59.66%, 1 trail) > Compound kushen (53.12%, 9 trails) > Placenta polypeptide (51.72%, 1 trail) > Shenmai (45.21%, 1 trail) > Xiaoaiping (36.56%, 2 trails) > Delisheng (36.52%, 1 trail) > Shenqifuzheng (26.47%, 5 trails).

**Table 2:** Results of the network meta-analysis for leukopenia (upper right quarter), gastrointestinal reaction (lower left quarter)

**AD +FOLFOX**	3.64 (2.03,6.65)	1.028 (0.22,4.54)	1.38 (0.40,4.71)	1.86 (0.81,4.14)	1.61 (0.51,5.10)	1.18 (0.57,2.47)	1.022 (0.31,3.35)	0.79 (0.19,3.13)	0.80 (0.35,1.77)	1.23 (0.30,4.68)	0.95 (0.23,4.01)	1.61 (0.54,4.61)	--	--
**2.74 (1.81,4.18)**	**FOLFOX**	0.28 (0.068,1.08)	0.38 (0.13,1.10)	**0.51 (0.28,0.88)**	0.44 (0.16,1.18)	**0.33 (0.21,0.49)**	**0.28 (0.099,0.77)**	**0.22 (0.060,0.74)**	**0.22 (0.12,0.37)**	0.33 (0.093,1.12)	**0.26 (0.069,0.94)**	0.44 (0.18,1.04)	--	--
0.82 (0.21,2.85)	**0.30 (0.082,0.98)**	**DC + FOLFOX**	1.33 (0.24,8.02)	1.82 (0.41,8.18)	1.57 (0.30,8.86)	1.16 (0.28,5.00)	0.99 (0.18,5.72)	0.76 (0.12,5.06)	0.77 (0.17,3.45)	1.18 (0.19,7.61)	0.94 (0.14,6.33)	1.55 (0.31,8.25)	--	--
0.77 (0.21,2.42)	**0.28 (0.083,0.80)**	0.94 (0.17,5.09)	**SM +FOLFOX**	1.36 (0.39,4.54)	1.17 (0.27,5.10)	0.86 (0.27,2.8)	0.74 (0.17,3.23)	0.57 (0.11,2.99)	0.58 (0.17,1.93)	0.88 (0.17,4.57)	0.69 (0.12,3.81)	1.16 (0.28,4.69)	--	--
1.004 (0.58,1.73)	**0.37 (0.26,0.52)**	1.23 (0.35,4.72)	1.31 (0.43,4.65)	**SQFZ + FOLFOX**	0.87 (0.28,2.79)	0.64 (0.32,1.32)	0.55 (0.17,1.80)	0.42 (0.11,1.68)	**0.43 (0.19,0.94)**	0.66 (0.17,2.55)	0.52 (0.12,2.12)	0.86 (0.30,2.46)	--	--
1.001 (0.38,2.62)	**0.37 (0.15,0.86)**	1.23 (0.28,5.92)	1.31 (0.34,5.76)	1.00 (0.39,2.52)	**DLS + FOLFOX**	0.73 (0.25,2.17)	0.63 (0.15,2.61)	0.49 (0.097,2.39)	0.49 (0.15,1.51)	0.76 (0.15,3.61)	0.60 (0.11,2.98)	0.99 (0.26,3.65)	--	--
1.24 (0.73,2.15)	**0.45 (0.32,0.64)**	1.53 (0.44,5.85)	1.63 (0.53,5.71)	1.24 (0.75,2.02)	1.25 (0.49,3.18)	**CKS + FOLFOX**	0.85 (0.28,2.59)	0.66 (0.17,2.48)	0.67 (0.33,1.32)	1.026 (0.27,3.76)	0.81 (0.20,3.15)	1.35 (0.50,3.54)	--	--
0.95 (0.45,1.99)	**0.35 (0.19,0.64)**	1.18 (0.30,5.02)	1.24 (0.37,4.78)	0.95 (0.47,1.92)	0.96 (0.33,2.76)	0.77 (0.37,1.55)	**HCS + FOLFOX**	0.77 (0.15,3.94)	0.78 (0.24,2.49)	1.21 (0.24,5.86)	0.94 (0.18,4.83)	1.58 (0.40,6.01)	--	--
0.44 (0.12,1.48)	**0.16 (0.049,0.50)**	0.54 (0.096,3.13)	0.58 (0.12,3.07)	0.44 (0.13,1.45)	0.44 (0.10,1.86)	0.35 (0.10,1.16)	0.45 (0.12,1.71)	**AP + FOLFOX**	1.009 (0.25,4.01)	1.56 (0.27,9.03)	1.23 (0.20,7.37)	2.042 (0.44,9.22)	--	--
1.18 (0.69,2.04)	**0.43 (0.31,0.6)**	1.46 (0.42,5.53)	1.55 (0.52,5.39)	1.18 (0.73,1.91)	1.19 (0.47,3.03)	0.95 (0.59,1.54)	1.24 (0.62,2.49)	2.72 (0.82,9.30)	**KA + FOLFOX**	1.53 (0.39,5.95)	1.21 (0.29,4.94)	2.011 (0.71,5.76)	--	--
0.80 (0.21,2.76)	**0.29 (0.082,0.94)**	0.97 (0.17,5.77)	1.051 (0.20,5.51)	0.79 (0.22,2.7)	0.79 (0.18,3.43)	0.64 (0.18,2.20)	0.83 (0.20,3.13)	1.79 (0.35,9.78)	0.68 (0.18,2.25)	**PL + FOLFOX**	0.79 (0.13,4.72)	1.31 (0.29,6.10)	--	--
1.096 (0.52,2.30)	**0.40 (0.21,0.74)**	1.35 (0.35,5.54)	1.44 (0.41,5.48)	1.094 (0.53,2.21)	1.097 (0.37,3.26)	0.89 (0.43,1.80)	1.15 (0.47,2.78)	2.52 (0.66,9.57)	0.92 (0.46,1.86)	1.37 (0.37,5.55)	**LE + FOLFO**X	1.66 (0.35,8.12)	--	--
1.96 (0.54,6.76)	0.71 (0.21,2.34)	2.42 (0.42,13.52)	2.57 (0.5,14.04)	1.95 (0.55,6.73)	1.96 (0.43,8.70)	1.57 (0.44,5.38)	2.04 (0.52,7.68)	4.42 (0.84,24.08)	1.64 (0.46,5.64)	2.46 (0.45,13.91)	1.79 (0.46,6.74)	**XAP + FOLFOX**	--	--
1.25 (0.34,4.77)	0.46 (0.13,1.61)	1.54 (0.28,9.59)	1.66 (0.33,9.30)	1.24 (0.34,4.60)	1.24 (0.28,5.90)	1.006 (0.28,3.76)	1.31 (0.34,5.28)	2.81 (0.55,16.15)	1.054 (0.30,3.86)	0.64 (0.11,3.40)	0.88 (0.21,3.44)	1.53 (0.26,9.29)	**EL + FOLFOX**	--
1.32 (0.36,4.53)	0.48 (0.14,1.53)	1.65 (0.29,9.15)	1.73 (0.34,9.11)	1.31 (0.37,4.38)	1.31 (0.30,5.59)	1.061 (0.30,3.56)	1.38 (0.36,5.16)	3.03 (0.57,15.64)	1.11 (0.31,3.71)	0.60 (0.11,3.28)	0.84 (0.22,3.29)	1.48 (0.28,7.96)	1.075 (0.18,5.48)	**GP +FOLFOX**

### Gastrointestinal reaction

57 eligible RCTs enrolling 14 types of CHIs reported the data of gastrointestinal reaction. In respect of gastrointestinal reaction, the results between comparisons were revealed in Table 2. As combination therapies, Aidi +FOLFOX (OR = 2.74, 95% CI = 1.81–4.18), Disodium cantharidinate and vitamin B6 +FOLFOX (OR = 0.30, 95% CI = 0.082–0.98), Shenmai +FOLFOX (OR = 0.28, 95% CI = 0.083–0.80), Shenqifuzheng +FOLFOX (OR = 0.37, 95% CI = 0.26–0.52), Delisheng +FOLFOX (OR = 0.37, 95% CI = 0.15–0.86), Compound kushen +FOLFOX (OR = 0.45, 95% CI = 0.32–0.64), Huachansu +FOLFOX (OR = 0.35, 95% CI = 0.19–0.64), Astragalus polysaccharides +FOLFOX (OR = 0.16, 95% CI = 0.049–0.50), Kangai +FOLFOX (OR = 0.43, 95% CI = 0.31–0.6), Placenta polypeptide +FOLFOX (OR = 0.29, 95% CI = 0.082–0.94), Lentinan +FOLFOX (OR = 0.40, 95% CI = 0.21–0.74) demonstrated significant superiority in relieving gastrointestinal reaction over FOLFOX regimen, and there were significant differences between these groups. Additionally, there was no significant difference between different CHIs groups in respect of gastrointestinal reaction. Given the cumulative probabilities for gastrointestinal reaction as presented in Figure 4B, 14 types of CHIs ranked as follows: Astragalus polysaccharides (88.56%, 1 trail) > Shenmai (68.16%, 1 trail) > Placenta polypeptide (65.5%, 1 trail) > Disodium cantharidinate and vitamin B6 (63.99%, 1 trail) > Huachansu (58.79%, 4 trails) > Shenqifuzheng (56.41%, 12 trails) > Aidi (56.29%, 8 trails) > Delisheng (54.7%, 1 trail) > Lentinan (48.95%, 4 trails) > Elemene (42.77%, 1 trail) > Kangai (41.83%, 12 trails) > Ginseng polysacchride (39.72%, 1 trail) > Compound kushen (38.05%, 9 trails) > Xiaoaiping (22.15%, 1 trails).

### Hepatic dysfunction

19 RCTs including 9 types of CHIs provided sufficient data for estimating hepatic dysfunction. The results showed that the combined therapies of Aidi +FOLFOX 4.38 (1.76,11.08), Shenqifuzheng +FOLFOX 0.15 (0.014,0.94), Compound kushen +FOLFOX 0.21 (0.11,0.38), Kangai +FOLFOX 0.34 (0.14,0.82) had significant benefits of relieving hepatic dysfunction compared with FOLFOX regimen, and statistical differences were detected between these groups. Among CHIs groups, there was no significantly statistical difference between groups (Table 3). According to Figure 4C, the rank of 9 CHIs based on cumulative probabilities for hepatic dysfunction was namely: Disodium cantharidinate and vitamin B6 (76.68%, 1 trail) > Shenqifuzheng (75.83%, 1 trail) > Compound kushen (70.54%, 6 trails) > Ginseng polysacchride (68.73%, 1 trail) > Aidi (66.51%, 3 trails) > Kangai (50.49%, 3 trails) > Xiaoaiping (30.99%, 2 trails) > Lentinan (24.62%, 1 trails) > Shenmai (22.26%, 1 trail). In addition, the SUCRA values of each comparison for 5 outcomes were summarized in Table 4.

**Table 3:** Results of the network meta-analysis for hepatic dysfunction (lower left quarter)

**AD+ FOLFOX**									
**4.38 (1.76,11.08)**	**FOLFOX**								
0.53 (0.016,6.48)	0.12 (0.0041,1.26)	**DC+ FOLFOX**							
3.69 (0.62,21.1)	0.84 (0.18,3.75)	7.01 (0.42,279.2)	**SM+ FOLFOX**						
0.64 (0.052,5.16)	**0.15 (0.014,0.94)**	1.19 (0.041,55.93)	0.17 (0.011,1.98)	**SQFZ+ FOLFOX**					
0.93 (0.30,2.72)	**0.21 (0.11,0.38)**	1.71 (0.15,52.25)	0.25 (0.048,1.28)	1.45 (0.20,16.1)	**CKS+ FOLFOX**				
1.51 (0.41,5.36)	**0.34 (0.14,0.82)**	2.78 (0.23,94.5)	0.41 (0.073,2.34)	2.39 (0.29,27.12)	1.63 (0.56,4.77)	**KA+ FOLFOX**			
0.86 (0.10,5.96)	0.20 (0.029,1.09)	1.60 (0.078,70.97)	0.23 (0.021,2.34)	1.37 (0.092,24.58)	0.93 (0.13,5.79)	0.57 (0.070,3.95)	**GP+ FOLFOX**		
4.17 (0.28,67.87)	0.96 (0.077,12.81)	8.066 (0.24,505.5)	1.13 (0.061,23.5)	6.94 (0.27,196.2)	4.54 (0.34,66.17)	2.79 (0.20,43.73)	5.035 (0.23,115.2)	**LE+ FOLFOX**	
2.70 (0.55,12.89)	0.61 (0.17,2.20)	5.095 (0.35,173.7)	0.73 (0.10,5.19)	4.27 (0.44,59.85)	2.90 (0.72,11.84)	1.79 (0.38,8.37)	3.13 (0.37,32.46)	0.64 (0.036,10.63)	**XAP+ FOLFOX**

**Table 4:** SUCRA values of 15 CHIs groups and FOLFOX group for 5 outcomes

	The clinical efficacy	Performance status	Leukopenia	Gastrointestinal reaction	Hepatic dysfunction
AD + FOLFOX	33.24%	28.68%	64.23%	56.29%	66.51%
FOLFOX	6.11%	0.76%	1.90%	4.11%	13.36%
AI + FOLFOX	83.45%	NR	NR	NR	NR
AP + FOLFOX	59.76%	97.12%	72.32%	88.56%	NR
CKS + FOLFOX	74.4%	37.26%	53.12%	38.05%	70.54%
DC + FOLFOX	NR	NR	59.66%	63.99%	76.68%
DLS + FOLFOX	NR	NR	36.52%	54.7%	NR
EL + FOLFOX	NR	44.85%	NR	42.77%	NR
GP + FOLFOX	27.72%	69.38%	NR	39.72%	68.73%
HCS + FOLFOX	53.01%	44.11%	61.36%	58.79%	NR
KA + FOLFOX	68.65%	71.23%	77.91%	41.83%	50.49%
LE + FOLFOX	65.87%	50.09%	63.02%	48.95%	24.62%
PL + FOLFOX	NR	NR	51.72%	65.5%	NR
SM + FOLFOX	NR	62.5%	45.21%	68.16%	22.26%
SQFZ + FOLFOX	42.14%	43.94%	26.47%	56.41%	75.83%
XAP + FOLFOX	35.66%	50.07%	36.56%	22.15%	30.99%

Note: NR:nor report; FOLFOX: FOLFOX Chemotherapy regimen; AD: Aidi injection; AI: Astragalus injection; AP: Astragalus polysaccharide injection; CKS: Compound kushen injection; DC: Disodium cantharidinate and vitamin B6 injection; DLS: Delisheng injection; EL: Elemene injection; GP: Ginseng Polysacchride Injection; HCS: Huachansu injection; KA: Kangai injection; LE: Lentinan injection; PL: Placenta polypeptide injection; SM: Shenmai injection; SQFZ: Shenqifuzheng injection; XAP: Xiaoaiping injection.

### Publication bias

**Figure 5:**
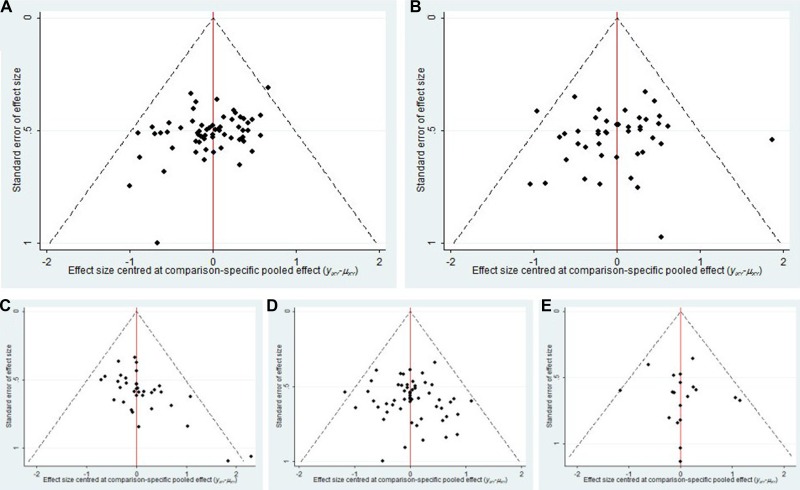
Funnel Plot for 5 outcomes Note: (**A**) Clinical efficacy; (**B**) Performance status; (**C**) Leucopenia; (**D**) Gastrointestinal reaction; (**E**) Hepatic dysfunction.

The publication bias of the included RCTs were measured by funnel plots. The funnel plots of 5 outcomes in this NMA illustrated there were potential publication bias among included RCTs (Figure 5).

### Cluster analysis

**Figure 6:**
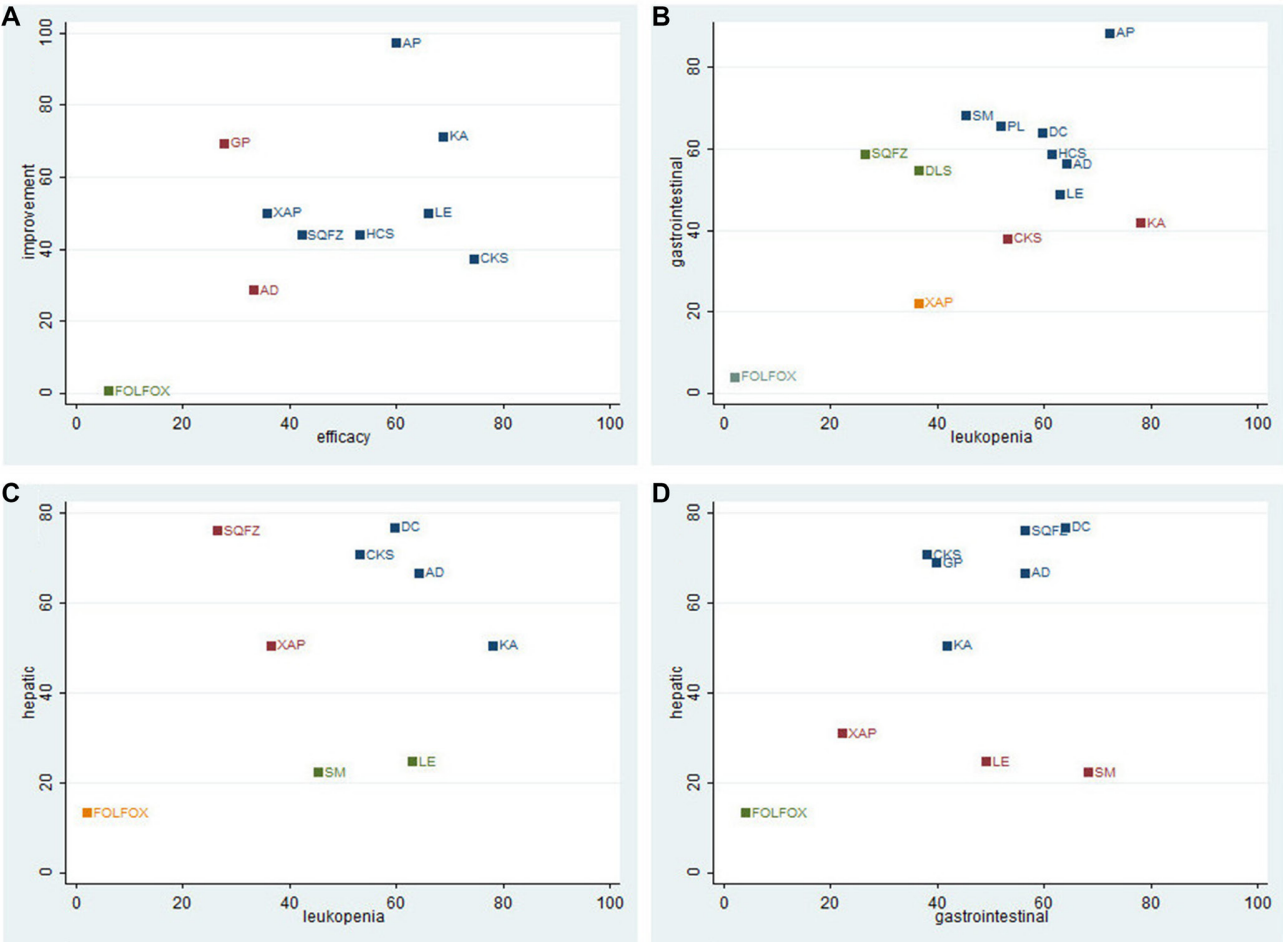
Cluster analysis Plot for 5outcomes Note: (**A**) Clinical efficacy (X axis) & Performance status (Y axis); (**B)** Leukopenia (X axis) & Gastrointestinal reaction (Y axis); (**C**) Leukopenia (X axis) & Hepatic dysfunction (Y axis); (**D**) Gastrointestinal reaction (X axis) & Hepatic dysfunction (Y axis); FOLFOX: FOLFOX Chemotherapy regimen; AD: Aidi injection; CKS: Compound kushen injection; SQFZ: Shenqifuzheng injection; HCS: Huachansu injection; XAP: Xiaoaipinginjection; LE: Lentinan injection; GP: Ginseng Polysacchride Injection; KA: Kangai injection; AP: Astragalus polysaccharide injection. DLS: Delisheng injection; DC: Disodium cantharidinate and vitamin B6 injection; PL: Placenta polypeptide injection; SM: Shenmai injection.

The cluster analysis based on SUCRA values indicated that interventions with same color belonged to the same cluster, and interventions located in the upper right corner seemed an optimal therapy for two different outcomes. The results of cluster analysis were revealed in Figure 6. First, a cluster analysis was conducted for 9 types of CHIs that reported both clinical efficacy, and performance status. Astragalus polysaccharides +FOLFOX and Kangai +FOLFOX showed a favorable improvement of clinical efficacy and performance status for patients with gastric cancer. While Aidi +FOLFOX was inferior in improving the primary outcomes among CHIs groups, and receiving FOLFOX regimen single was proved to be the worst intervention. Second, the results of cluster analysis which accessed 12 CHIs for leucopenia and gastrointestinal reaction indicated that Astragalus polysaccharides +FOLFOX was associated with a better effect on relieving both leucopenia and gastrointestinal reaction, by contrast, Xiaoaiping +FOLFOX exhibited an inferior response for these ADRs among CHIs groups. Third, 8 types of CHIs contributed into the cluster analysis for leucopenia and hepatic dysfunction. The therapies of Disodium cantharidinate and vitamin B6 +FOLFOX, Aidi +FOLFOX, Compound +FOLFOX were associated with preferable response in relieving leucopenia and hepatic dysfunction. Similarly, for gastrointestinal reaction and hepatic dysfunction, Disodium cantharidinate and vitamin B6 +FOLFOX also achieve superior effects for reducing both gastrointestinal reaction and hepatic dysfunction among CHIs groups.

## DISCUSSION

This NMA was conducted to compare 15 types of CHIs combined with FOLFOX regimen for the treatment of gastric cancer with regard to efficacy and safety. According to the results of this NMA, we suggested that Astragalus polysaccharides injection and Kangai injection combined with FOLFOX regimen were associated with preferable effects in improving clinical efficacy and performance status. In terms of ADRs, Astragalus polysaccharides injection and Disodium Cantharidinate and Vitamin B6 injection could achieve a favorable response in relieving ADRs. In general, different types of CHIs had different effects and functions on patients with gastric cancer [107]; therefore, the clinical decision of using CHIs should depend on the combination of doctors’ experience, patients’ condition and high-level evidence-based medical research.

In view of clinical manifestation, gastric cancer belonged to the category of “stomachache”, “dysphagia” and “nausea” in TCM theory [108]. Recently, TCM served as important parts of complementary and alternative medicine to provide a theoretical and practical approach to the treatment of gastric cancer combined with other conventional cancer therapies. And TCM was confirmed that it could not only improve the clinical symptoms, immune functions and performance status of cancer patients, but also prolong their survival period [109–110]. Moreover, Kangai injection was made from ginseng, Astragalus, Sophora flavescens, and its functions were replenishing Qi and strengthening the body resistance owing to its active components, namely Astragalus saponins, ginsenoside, and matrine. Correlative pharmacological studies reported that Kangai injection might influence the enzyme activities of macrophages and morphology for spleen and thymus from rats [111]. Meanwhile, Kangai injection combined with chemotherapy could achieve effects for treating malignant pleural effusion and for refractory/relapsed acute leukemia [112–113]. From the theory of TCM, the flavor of Astragalus mongholicus was sweet; the nature of it was slightly warm, and its actions included supplement and boost Qi and lifting Yang. Modern pharmacological studies also showed that different Astragali radix extracts had anti-neuropathic effects of oxaliplatin-induced neurotoxicity in a rat model [114]. On the one hand, Astragalus saponins could inhibit the growth both *in vitro* and vivo of human gastric cancer cells; on the other hand, Astragalus saponins were able to reduce the invasion ability and induce the apoptosis of gastric cancer cells [115]. In addition, it was proven that Astragalus polysaccharide can effectively alleviate inflammation and boost the immune system to achieve the strong effects of antitumor [116–117]. Meanwhile, some clinical studies revealed that Astragalus polysaccharide injection combined chemotherapy could improve the clinical efficacy and prolong the survival time of the patients with nonsmall-cell lung cancer [118].

FOLFOX regimen waas accepted as one of the first-line chemotherapy regimen for the treatment of gastric cancer internationally [7, 10]. The chemotherapy regimen of included RCTs was restricted to FOLFOX regimen for reducing the interference of clinical heterogeneity which was related to chemotherapeutic agents. Currently, there was one research of NMA regarding CHIs for treating gastric cancer [119], compared with this previous research, the advantages of this NMA were reflected in the following aspects: Firstly, the searching strategy was more comprehensive because that literature searches were conducted about 23 types of CHIs which were used for treating cancer at the present, and the searching strategy was amended and confirmed by experts on data retrieval methods. Besides, apart from searching electronic databases of Chinese and English, we also searched RCTs at related academic organization websites. Secondly, the inclusion and exclusion criteria were established strictly. The standardization of efficacy evaluation was only according to WHO criteria. Third, the results of our NMA was in accordance with the previous research in Astragalus polysaccharide injection could achieve a superior response for clinical efficacy and safety than other CHIs; however, with regard to the results of Kanglaite injection and Javanica oil emulsion injection, the conclusions were not totally consistent between this NMA and the previous research. Finally, we not only accessed the clinical efficacy, performance status and ADRs, but also evaluated the quality and estimated the publication bias of included RCTs; the cluster analysis was also conducted to select the best CHIs for different outcomes.

Also, this NMA faced several limitations inherent to the methodology applied. First of all, survival time was an important end-point outcome for evaluating the efficacy in the treatment of cancer. While only 4 RCTs in this NMA provided the sufficient data of survival time or follow-up as showed in Supplementary Table 1. Therefore, the data was not enough to perform a NMA for the outcome of survival time. Second, our results were limited by methodological quality of the included RCTs; the majority of included RCTs exhibited a high risk of bias, largely due to inadequate allocation concealment and blinding methods. Among 15 CHIs involved in this NMA, 6 types of CHIs only had 1 trial included. The trial bias might affect the comparison objectivity between the different CHIs. And there was lack of large sample-size trails and head-to-head comparisons which was conducted between different CHIs. Third, although CHIs were widely applied in China, the test population of CHI was subject to other countries and regions. And the included RCTs were conducted in China and published in Chinese; therefore, it is unclear whether the conclusions of our study apply to other populations. Given those shortcomings, our research were needed to confirm by large-sample and multicenter, head-to-head RCTs. Moreover, our research suggested that clinical trials should concentrate on improving methodological quality themselves, adopt more objective, international endpoints outcomes to provide more rigorous and reliable evidence for clinical decision-making. In spite of those limitations listed above, this NMA provided a clear rank and optimal options by comparing clinical efficacy, performance status, and ADRs of different CHIs combined with FOLFOX regimen for treating gastric cancer.

The results of this NMA suggested that among 15 types of CHIs, Astragalus polysaccharides injection combined with FOLFOX regimen seemed optimal for patients with gastric cancer in improving clinical efficacy and performance status, and relieving ADRs. However, our findings should be confirmed by more prospectively designed, large-sample and multi-center RCTs.

## MATERIALS AND METHODS

This systematic and NMA was conducted in accordance with Cochrane criteria and PRISMA guidelines.

### Search strategy

From inception to January 10, 2017, an extensive literature collection about RCTs regarding CHIs for treating gastric cancer was performed by comprehensive searching strategy. The electronic databases included PubMed, Embase, the Cochrane Library, and together with several Chinese databases: the China National Knowledge Infrastructure Database (CNKI), the Wan-Fang Database (WangFang), the Chinese Scientific Journals Full-text Database (VIP), and the Chinese Biomedical Literature Database (CBM). Literature management was conducted by using EndNote X7 software (Thomson Reuters Crp.3 Times Square, New York, The United States). The keywords about gastric cancer of PubMed yielded the following searching query: “Stomach Neoplasm, Stomach Neoplasm, Gastric Neoplasms, Gastric Neoplasm, Stomach Cancer*, Stomach Tumor*, Gastric Cancer*, Gastric Tumor*, Gastric Carcinoma, Stomach Carcinoma”. And the Chinese keywords about gastric cancer in this NMA were according to the mesh terms which were provided in CBM database. The specific search terms for each CHIs and retrieval strategies were shown in Supplementary Table 3.

### Selection criteria

Four researchers (DZ, JZ, MN, and WJ) took participate in making the inclusion and exclusion criteria of this NMA.

Only RCTs meeting the following criteria were included: (1) Participant: the included participants diagnosed as gastric cancer, and no gender, race, or nationality limitations were imposed. (2) Intervention/Control: The CHIs group was treated by CHIs combined with FOLFOX regimen, while FOLFOX group solely received FOLFOX regimen. The chemotherapeutic drugs of FOLFOX regimen included 5-Fu, LV and L-OHP. (3) Outcomes: The primary outcomes were the clinical efficacy and the performance status. The criterion of the rapeutical effect met the WHO for solid tumors released in 1979 [120]. The clinical efficacy was calculated as followings: the clinical efficacy = [number of complete response (CR) patients+ partial response (PR)]/ total number of patients × 100%. Performance status was assessed by KPS, patients were considered to improve performance status when their KPSs increased more than 10 points after treatment. The secondary outcomes were the ADRs involving the incidence of leucopenia, gastrointestinal reaction and hepatic dysfunction. And the criterion of the ADRs met the WHO for common toxicity criteria of chemotherapy drugs released in 1981 [121]. The incidence of ADRs = (number of patients occurred ADRs)/ total number of patients × 100%. (4) Study type: RCTs regarding CHIs were combined with FOLFOX regimen for the treatment of gastric cancer, with irrespective of blinding methods the publishing language.

The exclusion criteria were described as follows: (1) Participant: Patients were complicated by other cancers, contraindications of chemotherapy and obvious abnormalities in their electrocardiograms and liver and kidney functions. (2) Intervention/Control: The administration of CHIs was not intravenous infusion. And information about chemotherapeutic drugs, dose and duration of treatment was incomplete or incorrect. Except for FOLFOX regimen, patients also received by radiotherapy, hyperthermia, interventional therapy, etc. (3) Outcomes: there were not available data of clinical efficacy, performance status and ADRs, and the rapeutical effect or ADRs was not in accordance with the criterion of WHO. (4) Study type: the publication types were not RCTs; such as case reports, animal experiments, editorials, letters, and reviews. And for duplicated RCTs, only the most updated and comprehensive ones were chosen.

### Data extraction and quality assessment

The available data of included RCTs was extracted into a spreadsheet of Microsoft Excel (Microsoft Corp, Redmond, WA) by three researchers (DZ, JZ, MN), and two researchers (KW, XD) crosschecked the extracted data independently. The following items were extracted: (1) Study characteristics: title, authors’ names, publication year, and literature sources of RCTs; (2) Patient characteristics: the numbers, ages, genders, KPSs before treatment, tumor types, and tumor stages of patients; (3) Intervention information: the names, dosages, and treatment cycles of CHIs; (4) Outcomes: the measured data of clinical efficacy, performance status and ADRs. Additionally, more details about the product information of each CHI were presented in Supplementary Table 4.

Quality assessment (including the randomization method, follow-up, blind methods, allocation concealment, reasons for withdrawal, inclusion and exclusion criteria, adverse reactions, statistical methods, foundations, and medical ethics) was performed by two independent researchers (DZ, JW), and disagreements were resolved by consensus according to the Cochrane risk of bias tool [122] and the methodological section of the CONSORT statement [123].

### Statistical analysis

The odds ratios (OR) of dichotomous data was calculated to measure comparative efficacy and safety for each therapy, along with 95% confidence intervals (CI). All the results of OR value were presented as medians. STATA 12.0 software (Stata Corporation, College Station, TX, USA) was adopted to present all calculations and graphs of NMA, and Markov chain Monte Carlo methods were performed by Win-BUGS 1.4.3 software (MRC Biostatistics Unit, Cambridge, UK). First, the chi-squared test was used to evaluate heterogeneity among studies, and I^2^ was used to show the magnitude of this heterogeneity. Results of *P* ≥ 0.1 and I^2^ ≤ 50% suggested a lack of significant heterogeneity; in such cases, the Mantel-Haenszel fixed-effects model was chosen. For cases with *P* < 0.1 and I^2^ > 50%, the Mantel-Haenszel random-effects model should be applied [124–125]. Since taking into account the included trails differed methodologically and clinically, the random-effects model was introduced to perform this NMA[126]. Second, we used surface under the cumulative ranking probabilities (SUCRA) values to rank the examined treatments, with SUCRA values of 100% and 0% assigned to the best and worst treatments [127–128]. Third, publication bias was graphically accessed via a comparison-adjusted funnel plot. Furthermore, we utilized clustering methods and 2-dimensional plots to produce clusters of treatments. Evaluation of the inconsistency between direct and indirect comparisons was unnecessary because a loop connecting the three arms did not exist in this NMA.

## SUPPLEMENTARY MATERIALS AND TABLES











## Abbreviations

CHIs: Chinese herbal injections
RCTs: Randomized controlled trials
NMA: network meta-analysis
ADR: adverse reaction
NMA: network meta-analysis
OR: odds ratios
CI: confidence interval
5-Fu: 5-fluorouracil
LV: leucovorin
L-OHP: oxaliplatin
TCM: traditional Chinese medicine
CNKI: the China National Knowledge Infrastructure Database
VIP: the Chinese Scientific Journals Full-text Database
CBM: the Chinese Biomedical Literature Database
WHO: World Health Organization
KPS: Karnofsky performance score
SUCRA: surface under the cumulative ranking probabilities
AD: Aidi injection
DC: Disodium cantharidinate and vitamin B6 injection
SM: Shenmai injection
SQFZ: Shenqifuzheng injection
DLS: Delisheng injection
CKS: Compound kushen injection
HCS: Huachansu injection
AP: Astragalus polysaccharide injection
AI: Astragalus injection
KA: Kangai injection
EL: Elemene injection
GP: Ginseng Polysacchride injection
PL: Placenta polypeptide injection
LE: Lentinan injection
XAP: Xiaoaiping injection.
