# Supplementary material for: Comparative efficacy and safety of Chinese herbal injections combined with the FOLFOX regimen for treating gastric cancer in China: a network meta-analysis
Source: Oncotarget. 2017 Aug 18;8(40):68873–89. doi: 10.18632/oncotarget.20320 (PMC5620304; doi:10.18632/oncotarget.20320)
Supplement: Supplementary file 1 [file oncotarget-08-68873-s001.pdf]

# Comparative efficacy and safety of Chinese herbal injections combined with the FOLFOX regimen for treating gastric cancer in China: a network meta-analysis

## SUPPLEMENTARY MATERIALS

**Supplementary Table 1: The basic characteristics of the included RCTs.** See Supplementary_Table_1

**Supplementary Table 2: Quality evaluation of the included RCTs.** See Supplementary_Table_2

**Supplementary Table 3: Search strategy.** See Supplementary_Table_3

**Supplementary Table 4: More details about the product information of CHIs.** See Supplementary_Table_4
