# Supplementary material for: Comparative efficacy and safety of Chinese herbal injections combined with the FOLFOX regimen for treating gastric cancer in China: a network meta-analysis
Source: Oncotarget. 2017 Aug 18;8(40):68873–89. doi: 10.18632/oncotarget.20320 (PMC5620304; doi:10.18632/oncotarget.20320)
Supplement: Supplementary file 2 [file oncotarget-08-68873-s002.doc]

**Supplementary Table 1. The basic characteristics of the included RCTs**

| Study ID | Sex (M/F) | AVG  age | N(E/C) | TNM stages | EST (m) | KPS score | Therapy of experiment | Therapy of control | Treatment  (days) | Outcomes |
| --- | --- | --- | --- | --- | --- | --- | --- | --- | --- | --- |
| Wu T 2016[22] | 36/26 | 43.88 | 31/31 | NR | ≥6 | ≥60 | AD50-100mL+L-OHP+LV+5-Fu | L-OHP+LV+5-Fu | 21d×2 | ①③④ |
| Liao YQ 2011[23] | 65/19 | 62 | 41/43 | Ⅲ-Ⅳ | >6 | >60 | AD 50-80mL+L-OHP+LV+5-Fu | L-OHP+LV+5-Fu | 21d×2 | ①②③ |
| Zhang L 2014[24] | 35/29 | 59.25 | 32/32 | Ⅲ-Ⅳ | >3 | >60 | AD 50-80mL+L-OHP+LV+5-Fu | L-OHP+LV+5-Fu | 21d×2 | ①③ |
| Chen NJ 2008[25] | 38/32 | 56.7 | 36/34 | Ⅲ-Ⅳ | NR | >70 | AD 50mL+L-OHP+LV+5-Fu | L-OHP+LV+5-Fu | 14d×3 | ①②③ |
| Song SJ 2014[26] | 26/24 | 55.6 | 25/25 | Ⅲ-Ⅳ | NR | NR | AD 50mL+L-OHP+LV+5-Fu | L-OHP+LV+5-Fu | 14d×3 | ① |
| He CS 2012[27] | 43/22 | 48.5 | 33/32 | NR | NR | NR | AD 50mL+L-OHP+LV+5-Fu | L-OHP+LV+5-Fu | 21d×2 | ②③ |
| Yan HX 2012[28] | 57/9. | 61.7 | 34/32 | Ⅲ-Ⅳ | NR | >70 | AD 100mL+L-OHP+LV+5-Fu | L-OHP+LV+5-Fu | 14d | ①③ |
| Zhang AX 2009[29] | 39/28 | 54 | 35/32 | NR | NR | NR | AD 50mL+L-OHP+LV+5-Fu | L-OHP+LV+5-Fu | 14d×3 | ②③ |
| Zhang MJ 2014[30] | 54/42 | 55.9 | 48/48 | Ⅲ-Ⅳ | NR | ≥60 | AD 50mL+L-OHP+LV+5-Fu | L-OHP+LV+5-Fu | 21d×2 | ①③ |
| Li GP 2010[31] | 37/13 | 40-65 | 25/25 | Ⅱ-Ⅳ | NR | >60 | DC 40m1+L-OHP+LV+5-Fu | L-OHP+LV+5-Fu | 14d×(3-4) | ③ |
| Zhang SQ 2013[32] | NR | NR | 46/45 | NR | NR | NR | SM 40mL+L-OHP+LV+5-Fu | L-OHP+LV+5-Fu | 14d×4 | ②③ |
| Song SQ 2015[33] | 16/14 | 51.3 | 15/15 | Ⅱ-Ⅲ | >3 | >60 | SQFZ 250ml+L-OHP+LV+5-Fu | L-OHP+LV+5-Fu | 14d | ③ |
| Liu H 2011[34] | 46/39 | 64.9 | 45/40 | Ⅲ-Ⅳ | >6 | ≥60 | SQFZ 250ml+L-OHP+LV+5-Fu | L-OHP+LV+5-Fu | 14d×2 | ② |
| Xie YF 2015[35] | 38/22 | 63.2 | 30/30 | Ⅱ-Ⅲ | >3 | >60 | SQFZ 250ml+L-OHP+LV+5-Fu | L-OHP+LV+5-Fu | 14d×2 | ③ |
| Xu XG 2015[36] | 41/21 | 56.6 | 31/31 | Ⅲ-Ⅳ | >5 | >60 | SQFZ 250ml+L-OHP+LV+5-Fu | L-OHP+LV+5-Fu | 21d | ① |
| Pa TM 2012[37] | 39/33 | 26-75 | 37/35 | NR | >3 | ≥60 | SQFZ 250ml+L-OHP+LV+5-Fu | L-OHP+LV+5-Fu | 28d×4 | ①② |
| Wang M 2011[38] | 43/37 | 50.5 | 40/40 | NR | NR | NR | SQFZ 250ml+L-OHP+LV+5-Fu | L-OHP+LV+5-Fu | 14d×4 | ① |
| Fang XY 2010[39] | 37/33 | 56.7 | 36/34 | Ⅲ-Ⅳ | NR | >70 | SQFZ 250ml+L-OHP+LV+5-Fu | L-OHP+LV+5-Fu | 14d×4 | ①②③ |
| Jia JW 2009[40] | 27/21 | 52.5 | 24/24 | NR | >3 | ≥60 | SQFZ 250ml+L-OHP+LV+5-Fu | L-OHP+LV+5-Fu | 14d×4 | ①②③ |
| Wang P 2014[41] | 45/39 | 65 | 42/42 | Ⅳ | ≥3 | >70 | SQFZ 250ml+L-OHP+LV+5-Fu | L-OHP+LV+5-Fu | 14d×2 | ①③ |
| Ren YZ 2012[42] | 30/35 | 62 | 33/32 | Ⅳ | ≥6 | >70 | SQFZ 250ml+L-OHP+LV+5-Fu | L-OHP+LV+5-Fu | 14d | ①③ |
| Sun YF 2008[43] | 98/26 | 56 | 60/64 | NR | NR | 60-70 | SQFZ 250ml+L-OHP+LV+5-Fu | L-OHP+LV+5-Fu | 21d×2 | ①②③ |
| Wen J 2014[44] | 23/7. | 59.8 | 15/15 | Ⅲ-Ⅳ | >3 | ≥60 | SQFZ 250ml+L-OHP+LV+5-Fu | L-OHP+LV+5-Fu | 14d×4 | ②③ |
| Li HY 2013[45] | 35/35 | 65.2 | 35/35 | Ⅲ-Ⅳ | >3 | ≥70 | SQFZ 250ml+L-OHP+LV+5-Fu | L-OHP+LV+5-Fu | 14d×4 | ①②③ |
| Lai CH 2013[46] | 39/18 | 58 | 29/28 | NR | >3 | ≥70 | SQFZ 250ml+L-OHP+LV+5-Fu | L-OHP+LV+5-Fu | 14d×2 | ①②③ |
| Chen LL 2012[47] | 43/27 | 49.5 | 35/35 | NR | NR | NR | SQFZ 250ml+L-OHP+LV+5-Fu | L-OHP+LV+5-Fu | 14d×4 | ①②③ |
| Zhu LF 2007[48] | 98/50 | 50.6 | 88/60 | Ⅱ-Ⅳ | NR | NR | SQFZ 250ml+L-OHP+LV+5-Fu | L-OHP+LV+5-Fu | 28d×6 | ② |
| Wang LX 2006[49] | NR | 32-70 | 37/30 | Ⅲ-Ⅳ | >2 | NR | SQFZ 250ml+L-OHP+LV+5-Fu | L-OHP+LV+5-Fu | 14d×2 | ①③ |
| Huang D 2014[50] | 26/22 | 52.3 | 24/24 | NR | NR | NR | SQFZ 250ml+L-OHP+LV+5-Fu | L-OHP+LV+5-Fu | 14d×2 | ①③ |
| He ZQ 2008[51] | 68/55 | 58.5 | 65/58 | Ⅳ | >3 | >60 | DLS 40ml+L-OHP+LV+5-Fu | L-OHP+LV+5-Fu | 20d | ③ |
| Lu XF 2015[52] | 68/23 | 44.8 | 46/45 | NR | NR | NR | CKS 20mL+L-OHP+LV+5-Fu | L-OHP+LV+5-Fu | 15d×4 | ② |
| Lu XY 2012[53] | 32/31 | 59.5 | 33/30 | Ⅲ-Ⅳ | ≥3 | NR | CKS 20mL+L-OHP+LV+5-Fu | L-OHP+LV+5-Fu | 21d | ③ |
| Zhang LQ 2010[54] | 39/29 | 54.8 | 34/34 | Ⅱ-Ⅲ | NR | ≥70 | CKS 20mL+L-OHP+LV+5-Fu | L-OHP+LV+5-Fu | 28d×6 | ①③ |
| Qin HB 2012[55] | 35/13 | 54.5 | 27/21 | Ⅲ-Ⅳ | >3 | NR | CKS 20mL+L-OHP+LV+5-Fu | L-OHP+LV+5-Fu | 21d×2 | ①③ |
| Liu KH 2014[56] | 86/66 | 59.5 | 77/75 | Ⅲ-Ⅳ | >3 | ≥60 | CKS 20mL+L-OHP+LV+5-Fu | L-OHP+LV+5-Fu | 28d×4 | ①②③ |
| Zhao Y 2011[57] | 31/9. | 53.2 | 20/20 | Ⅲ-Ⅳ | >3 | ≥50 | CKS 20mL+L-OHP+LV+5-Fu | L-OHP+LV+5-Fu | 21d×3 | ①②③ |
| Luo W 2014[58] | 15/3. | 55.4 | 9/9. | Ⅰ-Ⅱ | NR | >60 | CKS 20mL+L-OHP+LV+5-Fu | L-OHP+LV+5-Fu | 14d×2 | ①② |
| Han QL 2011[59] | 44/34 | 54.4 | 39/39 | Ⅲ-Ⅳ | NR | NR | CKS 15mL+L-OHP+LV+5-Fu | L-OHP+LV+5-Fu | 14d×2 | ①③ |
| Liu SL 2009[60] | 38/19 | 55.8 | 29/28 | NR | >3 | 60-90 | CKS 20mL+L-OHP+LV+5-Fu | L-OHP+LV+5-Fu | 21d×2 | ①③ |
| Song SJ 2013[61] | NR | NR | 40/40 | Ⅲ-Ⅳ | >3 | >60 | CKS 15mL+L-OHP+LV+5-Fu | L-OHP+LV+5-Fu | 14d×2 | ①③ |
| Feng XM 2013[62] | NR | NR | 52/52 | Ⅲ-Ⅳ | NR | NR | CKS 20mL+L-OHP+LV+5-Fu | L-OHP+LV+5-Fu | 21d | ①③ |
| Yang XE 2013[63] | 52/48 | 56.5 | 50/50 | Ⅲ-Ⅳ | ≥3 | NR | CKS 30mL+L-OHP+LV+5-Fu | L-OHP+LV+5-Fu | 21d | ①② |
| Huang ZF 2009[64] | 46/14 | 45.7 | 30/30 | Ⅱ-Ⅳ | ≥3 | ≥60 | CKS 20mL+L-OHP+LV+5-Fu | L-OHP+LV+5-Fu | 14d×2 | ①②③ |
| Yang JW 2012[65] | 30/28 | 64.1 | 30/28 | Ⅱ-Ⅳ | ≥3 | ≥60 | CKS 15mL+L-OHP+LV+5-Fu | L-OHP+LV+5-Fu | 14d×6 | ① |
| Chen XQ 2010[66] | 68/50 | 51.3 | 62/56 | Ⅰ-Ⅲ | NR | ≥70 | CKS 15mL+L-OHP+LV+5-Fu | L-OHP+LV+5-Fu | 14d×(8-12) | ③ |
| Liu YH 2010[67] | 118/48 | 72 | 83/83 | Ⅱ-Ⅳ | NR | NR | CKS 20mL+L-OHP+LV+5-Fu | L-OHP+LV+5-Fu | 21d×4 | ① |
| Xu JX 2013[68] | 74/46 | 18-75 | 60/60 | Ⅲ-Ⅳ | ≥3 | ≥60 | CKS 15mL+L-OHP+LV+5-Fu | L-OHP+LV+5-Fu | 21d×(2-6) | ②③ |
| Zhang Y 2005[69] | NR | 57 | 28/29 | Ⅳ | NR | ≥40 | HCS 50mL+L-OHP+LV+5-Fu | L-OHP+LV+5-Fu | 21d×3 | ①②③④ |
| Wang ZF 2012[70] | 39/9. | 58.9 | 24/24 | NR | >3 | >60 | HCS10-20mL+L-OHP+LV+5-Fu | L-OHP+LV+5-Fu | 14d×8 | ①② |
| Lu CH 2014[71] | 34/28 | 54 | 31/31 | NR | NR | NR | HCS 20mL+L-OHP+LV+5-Fu | L-OHP+LV+5-Fu | 21d×3 | ① |
| Cui P 2009[72] | NR | 53 | 32/22 | Ⅳ | NR | NR | HCS 30mL+L-OHP+LV+5-Fu | L-OHP+LV+5-Fu | 21d×2 | ①②③ |
| Wang YH 2009[73] | 48/20 | 54 | 36/32 | Ⅲ-Ⅳ | >3 | >60 | HCS 20mL+L-OHP+LV+5-Fu | L-OHP+LV+5-Fu | 28d×4 | ①③ |
| Wang WM2010[74] | 27/16 | 52 | 20/23 | NR | >3 | >60 | HCS10-20mL+L-OHP+LV+5-Fu | L-OHP+LV+5-Fu | 14d×8 | ①③ |
| Guo HR 2012[75] | 39/33 | 52 | 36/36 | NR | >3 | ≥60 | AI 50ml+L-OHP+LV+5-Fu | L-OHP+LV+5-Fu | 14d | ① |
| Lai YB 2014[76] | 47/33 | 49.3 | 40/40 | NR | NR | NR | AP 250mg+L-OHP+LV+5-Fu | L-OHP+LV+5-Fu | 21d | ② |
| Liu YH 2011[77] | 38/27 | 56 | 33/32 | Ⅰ-Ⅳ | NR | ≥60 | AP 250mg+L-OHP+LV+5-Fu | L-OHP+LV+5-Fu | 21d×4 | ①②③ |
| Li SQ 2015[78] | 33/17 | 66.4 | 25/25 | Ⅳ | >3 | >70 | KA 40ml+L-OHP+LV+5-Fu | L-OHP+LV+5-Fu | 21d×2 | ①②③ |
| Yang XM 2012[79] | 50/30 | 66.4 | 40/40 | Ⅳ | >3 | ≥70 | KA 40ml+L-OHP+LV+5-Fu | L-OHP+LV+5-Fu | 21d×2 | ①②③ |
| Zhou WJ 2015[80] | 93/65 | 73.8 | 80/78 | Ⅳ | >3 | ≥70 | KA 40ml+L-OHP+LV+5-Fu | L-OHP+LV+5-Fu | 28d×2 | ②③ |
| Li YY 2008[81] | 55/35 | 18-74 | 48/42 | NR | >3 | ≥70 | KA 40ml+L-OHP+LV+5-Fu | L-OHP+LV+5-Fu | 14d×(3-4) | ②③ |
| Qi YJ 2008[82] | 41/23 | 46.5 | 32/32 | NR | >3 | NR | KA 40ml+L-OHP+LV+5-Fu | L-OHP+LV+5-Fu | 14d×2 | ①③ |
| Wang JH 2011[83] | 34/15 | 63.2 | 24/25 | NR | NR | NR | KA 40ml+L-OHP+LV+5-Fu | L-OHP+LV+5-Fu | 28d×4 | ①③ |
| Mo YY 2010[84] | 46/30 | 56.3 | 40/36 | Ⅲ-Ⅳ | >3 | ≥60 | KA 60ml+L-OHP+LV+5-Fu | L-OHP+LV+5-Fu | 21d×2 | ①②③ |
| Wang LJ 2008[85] | 68/12. | 53 | 42/38 | NR | NR | >60 | KA 40ml+L-OHP+LV+5-Fu | L-OHP+LV+5-Fu | 21d×2 | ①②③ |
| Wu L 2008[86] | 54/26 | 31-78 | 40/40 | Ⅲ-Ⅳ | >3 | ≥60 | KA 50ml+L-OHP+LV+5-Fu | L-OHP+LV+5-Fu | 30d×2 | ①②③ |
| Wang XQ 2013[87] | 58/22 | 53 | 40/40 | NR | NR | NR | KA 30ml+L-OHP+LV+5-Fu | L-OHP+LV+5-Fu | 30d | ①②③ |
| Jiang L 2013[88] | 41/19 | 58.3 | 30/30 | Ⅲ-Ⅳ | >3 | ≥60 | KA 40ml+L-OHP+LV+5-Fu | L-OHP+LV+5-Fu | 14d×4 | ①④ |
| Huang PJ 2013[89] | 54/41 | 67.8 | 50/45 | Ⅲ-Ⅳ | NR | NR | KA 40ml+L-OHP+LV+5-Fu | L-OHP+LV+5-Fu | 14d×3 | ①③ |
| Leng S 2015[90] | 69/59 | 67.9 | 64/64 | NR | >3 | NR | KA 40ml+L-OHP+LV+5-Fu | L-OHP+LV+5-Fu | 14d×(3-4) | ① |
| Su XH 2013[91] | 38/22 | 72.1 | 30/30 | NR | NR | NR | KA 50ml+L-OHP+LV+5-Fu | L-OHP+LV+5-Fu | 28d×4 | ①③ |
| Zeng DX 2011[92] | 28/22 | 52.5 | 25/24 | Ⅲ-Ⅳ | >3 | ≥70 | EL 100ml+L-OHP+LV+5-Fu | L-OHP+LV+5-Fu | 14d | ②③ |
| Xu JD 2015[93] | 32/36 | 58 | 35/33 | Ⅳ | >3 | >70 | GP 12mg+L-OHP+LV+5-Fu | L-OHP+LV+5-Fu | 14d×2 | ①②③ |
| Zhang JX2011[94] | 36/27 | 55 | 38/25 | NR | >3 | ≥60 | GP 24mg+L-OHP+LV+5-Fu | L-OHP+LV+5-Fu | 14d×3 | ①② |
| Zhao LL 2014[95] | 37/25 | 18-75 | 31/31 | Ⅲ-Ⅳ | NR | ≥60 | PL 8mL+L-OHP+LV+5-Fu | L-OHP+LV+5-Fu | 14d | ③ |
| Chang YF2008[96] | 47/16 | 72.6 | 32/32 | Ⅲ-Ⅳ | >3 | ≥60 | LE 1mg+L-OHP+LV+5-Fu | L-OHP+LV+5-Fu | (21-28)d×4 | ①②③④ |
| Wu ZY 2013[97] | NR | 61.5 | 21/21 | Ⅲ-Ⅳ | >3 | ≥60 | LE 1mg+L-OHP+LV+5-Fu | L-OHP+LV+5-Fu | 14d×3 | ①②③ |
| WangHM2009[98] | 42/26 | 57 | 34/34 | NR | >3 | ≥70 | LE 1mg+L-OHP+LV+5-Fu | L-OHP+LV+5-Fu | 14d×2 | ①②③ |
| Li XL 2015[99] | 48/38 | 56 | 43/43 | Ⅳ | >3 | ≥70 | LE 12mg+L-OHP+LV+5-Fu | L-OHP+LV+5-Fu | 14d×2 | ①② |
| Li YY 2010[100] | 51/23 | 23-76 | 36/38 | NR | >3 | ≥70 | LE 1mg+L-OHP+LV+5-Fu | L-OHP+LV+5-Fu | 14d×3 | ②③ |
| Sai FD 2012[101] | 52/16 | 54.5 | 33/35 | Ⅲ-Ⅳ | >3 | ≥60 | XAP 60mg+L-OHP+LV+5-Fu | L-OHP+LV+5-Fu | 14d×4 | ①②③ |
| Liu WD 2012[102] | 31/15 | 51 | 28/28 | NR | >3 | ≥70 | XAP 80ml+L-OHP+LV+5-Fu | L-OHP+LV+5-Fu | 14d×4 | ①②③ |

Note: M:male; F:female; E:experimental group; C:control group: NR: no reported; EST: expected survival time; m: month; ①: clinical efficacy; ②: performance status; ③: ADR；④：survival rate; L-OHP: oxaliplatin; LV: leucovorin; 5-FU: 5-Fluorouracil; AD: Aidi injection; DC: Disodium cantharidinate and vitamin B6 injection; SM: Shenmai injection; SQFZ: Shenqifuzheng injection; DLS: Delisheng injection; CKS: Compound kushen injection; HCS: Huachansu injection; AP: Astragalus polysaccharide injection; AI: Astragalus injection; KA: Kangai injection; EL: Elemene injection; GP: Ginseng Polysacchride injection; PL: Placenta polypeptide injection; LE: Lentinan injection; XAP: Xiaoaiping injection.
