# Supplementary material for: Comparative efficacy and safety of Chinese herbal injections combined with the FOLFOX regimen for treating gastric cancer in China: a network meta-analysis
Source: Oncotarget. 2017 Aug 18;8(40):68873–89. doi: 10.18632/oncotarget.20320 (PMC5620304; doi:10.18632/oncotarget.20320)
Supplement: Supplementary file 3 [file oncotarget-08-68873-s003.doc]

**Supplmentary Table 2: Quality Evaluation of the Included RCTs**

| Study ID | Randomized method | Follow-up | Blind method | Allocation concealment | Reasons of withdrawal | Inclusion and exclusion criteria | ADRs | Statistical methods | Foundations | Medicalethics | Scores |
| --- | --- | --- | --- | --- | --- | --- | --- | --- | --- | --- | --- |
| Wu T 2016 | 0 | 1 | 0 | 0 | 0 | 1 | 1 | 1 | 1 | 1 | 6 |
| Liao YQ 2011 | 0 | 0 | 0 | 0 | 0 | 1 | 1 | 1 | 0 | 0 | 3 |
| Zhang L 2014 | 0 | 0 | 0 | 0 | 0 | 1 | 1 | 1 | 0 | 0 | 3 |
| Chen NJ 2008 | 0 | 0 | 0 | 0 | 0 | 1 | 1 | 1 | 0 | 0 | 3 |
| Song SJ 2014 | 0 | 0 | 0 | 0 | 0 | 1 | 0 | 1 | 0 | 1 | 3 |
| He CS 2012 | 1 | 0 | 0 | 0 | 0 | 1 | 1 | 1 | 0 | 0 | 4 |
| Yan HX 2012 | 0 | 0 | 0 | 0 | 0 | 1 | 1 | 1 | 0 | 1 | 4 |
| Zhang AX 2009 | 0 | 0 | 0 | 0 | 0 | 1 | 1 | 1 | 0 | 0 | 3 |
| Zhang MJ 2014 | 0 | 1 | 0 | 0 | 0 | 1 | 1 | 1 | 0 | 1 | 5 |
| Li GP 2010 | 0 | 0 | 0 | 0 | 0 | 1 | 1 | 1 | 0 | 0 | 3 |
| Zhang SQ 2013 | 0 | 0 | 0 | 0 | 0 | 1 | 1 | 1 | 0 | 0 | 3 |
| Song SQ 2015 | 0 | 1 | 0 | 0 | 0 | 1 | 1 | 1 | 0 | 0 | 4 |
| Liu H 2011 | 0 | 0 | 0 | 0 | 0 | 1 | 0 | 1 | 0 | 0 | 2 |
| Xie YF 2015 | 1 | 0 | 0 | 0 | 0 | 1 | 1 | 1 | 1 | 1 | 6 |
| Xu XG 2015 | 0 | 0 | 0 | 0 | 0 | 1 | 0 | 1 | 0 | 0 | 2 |
| Pa TM 2012 | 0 | 0 | 0 | 0 | 0 | 1 | 0 | 1 | 0 | 1 | 3 |
| Wang M 2011 | 0 | 0 | 0 | 0 | 0 | 1 | 0 | 1 | 0 | 0 | 2 |
| Fang XY 2010 | 0 | 0 | 0 | 0 | 0 | 1 | 1 | 1 | 0 | 0 | 3 |
| Jia JW 2009 | 0 | 0 | 0 | 0 | 0 | 1 | 1 | 1 | 0 | 0 | 3 |
| Wang P 2014 | 0 | 0 | 0 | 0 | 0 | 1 | 1 | 1 | 0 | 1 | 4 |
| Ren YZ 2012 | 0 | 0 | 1 | 0 | 0 | 1 | 1 | 1 | 0 | 1 | 5 |
| Sun YF 2008 | 0 | 0 | 0 | 0 | 0 | 1 | 1 | 1 | 0 | 0 | 3 |
| Wen J 2014 | 0 | 0 | 0 | 0 | 0 | 1 | 1 | 1 | 0 | 0 | 3 |
| Li HY 2013 | 0 | 0 | 0 | 0 | 0 | 1 | 1 | 1 | 0 | 1 | 4 |
| Lai CH 2013 | 0 | 0 | 0 | 0 | 0 | 1 | 1 | 1 | 0 | 0 | 3 |
| Chen LL 2012 | 0 | 0 | 0 | 0 | 0 | 1 | 1 | 1 | 0 | 0 | 3 |
| Zhu LF 2007 | 0 | 0 | 0 | 0 | 0 | 1 | 0 | 1 | 0 | 0 | 2 |
| Wang LX 2006 | 0 | 0 | 0 | 0 | 0 | 1 | 1 | 1 | 0 | 0 | 3 |
| Huang D 2014 | 0 | 0 | 0 | 0 | 0 | 1 | 1 | 1 | 0 | 0 | 3 |
| He ZQ 2008 | 1 | 1 | 0 | 0 | 0 | 1 | 1 | 1 | 0 | 0 | 5 |
| Lu XF 2015 | 0 | 0 | 0 | 0 | 0 | 1 | 0 | 1 | 0 | 1 | 3 |
| Lu XY 2012 | 0 | 0 | 0 | 0 | 0 | 1 | 1 | 1 | 0 | 0 | 3 |
| Zhang LQ 2010 | 0 | 0 | 0 | 0 | 0 | 1 | 1 | 1 | 0 | 0 | 3 |
| Qin HB 2012 | 0 | 0 | 0 | 0 | 0 | 1 | 1 | 1 | 1 | 0 | 4 |
| Liu KH 2014 | 0 | 0 | 0 | 0 | 0 | 1 | 1 | 1 | 1 | 1 | 5 |
| Zhao Y 2011 | 0 | 0 | 0 | 0 | 0 | 1 | 1 | 1 | 0 | 0 | 3 |
| Luo W 2014 | 1 | 0 | 0 | 0 | 0 | 1 | 0 | 1 | 0 | 0 | 3 |
| Han QL 2011 | 0 | 0 | 0 | 0 | 0 | 1 | 1 | 1 | 0 | 0 | 3 |
| Liu SL 2009 | 0 | 0 | 0 | 0 | 0 | 1 | 1 | 1 | 0 | 0 | 3 |
| Song SJ 2013 | 1 | 0 | 0 | 0 | 0 | 1 | 1 | 1 | 0 | 1 | 5 |
| Feng XM 2013 | 0 | 0 | 0 | 0 | 0 | 1 | 1 | 1 | 0 | 0 | 3 |
| Yang XE 2013 | 0 | 0 | 0 | 0 | 0 | 1 | 0 | 1 | 0 | 0 | 2 |
| Huang ZF 2009 | 1 | 0 | 0 | 0 | 0 | 1 | 1 | 1 | 0 | 0 | 4 |
| Yang JW 2012 | 0 | 0 | 0 | 0 | 0 | 1 | 0 | 1 | 0 | 0 | 2 |
| Chen XQ 2010 | 0 | 0 | 0 | 0 | 0 | 1 | 1 | 1 | 0 | 0 | 3 |
| Liu YH 2010 | 0 | 0 | 0 | 0 | 0 | 1 | 0 | 1 | 0 | 0 | 2 |
| Xu JX 2013 | 0 | 0 | 0 | 0 | 0 | 1 | 1 | 1 | 0 | 1 | 4 |
| Zhang Y 2005 | 0 | 1 | 0 | 0 | 0 | 1 | 1 | 1 | 0 | 0 | 4 |
| Wang ZF 2012 | 1 | 0 | 0 | 0 | 0 | 1 | 0 | 1 | 0 | 0 | 3 |
| Lu CH 2014 | 0 | 0 | 0 | 0 | 0 | 1 | 1 | 1 | 0 | 0 | 3 |
| Cui P 2009 | 0 | 0 | 0 | 0 | 0 | 1 | 1 | 1 | 0 | 0 | 3 |
| Wang YH 2009 | 0 | 0 | 0 | 0 | 0 | 1 | 1 | 1 | 0 | 0 | 3 |
| Wang WM 2010 | 0 | 1 | 0 | 0 | 0 | 1 | 1 | 1 | 0 | 0 | 4 |
| Guo HR 2012 | 0 | 0 | 0 | 0 | 0 | 1 | 0 | 1 | 0 | 0 | 2 |
| Lai YB 2014 | 0 | 0 | 0 | 0 | 0 | 1 | 0 | 1 | 0 | 1 | 3 |
| Liu YH 2011 | 0 | 0 | 0 | 0 | 0 | 1 | 1 | 1 | 0 | 0 | 3 |
| Li SQ 2015 | 1 | 0 | 0 | 0 | 0 | 1 | 1 | 1 | 0 | 0 | 4 |
| Yang XM 2012 | 1 | 0 | 0 | 0 | 0 | 1 | 1 | 1 | 0 | 1 | 5 |
| Zhou WJ 2015 | 1 | 0 | 0 | 0 | 0 | 1 | 1 | 1 | 0 | 1 | 5 |
| Li YY 2008 | 0 | 0 | 0 | 0 | 0 | 1 | 1 | 1 | 0 | 0 | 3 |
| Qi YJ 2008 | 0 | 0 | 0 | 0 | 0 | 1 | 1 | 1 | 0 | 0 | 3 |
| Wang JH 2011 | 0 | 0 | 0 | 0 | 0 | 1 | 1 | 1 | 0 | 0 | 3 |
| Mo YY 2010 | 0 | 0 | 0 | 0 | 0 | 1 | 1 | 1 | 0 | 0 | 3 |
| Wang LJ 2008 | 0 | 0 | 0 | 0 | 0 | 1 | 1 | 1 | 0 | 0 | 3 |
| Wu L 2008 | 0 | 0 | 0 | 0 | 0 | 1 | 1 | 1 | 0 | 0 | 3 |
| Wang XQ 2013 | 1 | 0 | 0 | 0 | 0 | 1 | 1 | 1 | 0 | 0 | 4 |
| Jiang L 2013 | 0 | 1 | 0 | 0 | 0 | 1 | 0 | 1 | 0 | 0 | 3 |
| Huang PJ 2013 | 0 | 0 | 0 | 0 | 0 | 1 | 1 | 1 | 0 | 1 | 4 |
| Leng S 2015 | 1 | 0 | 0 | 0 | 0 | 1 | 0 | 1 | 0 | 1 | 4 |
| Su XH 2013 | 0 | 0 | 0 | 0 | 0 | 1 | 1 | 1 | 0 | 0 | 3 |
| Zeng DX 2011 | 0 | 0 | 0 | 0 | 0 | 1 | 1 | 1 | 0 | 1 | 4 |
| Xu JD 2015 | 0 | 0 | 1 | 0 | 0 | 1 | 1 | 1 | 0 | 1 | 5 |
| Zhang JX 2011 | 0 | 0 | 0 | 0 | 0 | 1 | 0 | 1 | 0 | 0 | 2 |
| Zhao LL 2014 | 1 | 0 | 0 | 0 | 0 | 1 | 1 | 1 | 1 | 1 | 6 |
| Chang YF 2008 | 0 | 1 | 0 | 0 | 0 | 1 | 1 | 1 | 0 | 0 | 4 |
| Wu ZY 2013 | 1 | 0 | 0 | 0 | 0 | 1 | 1 | 1 | 0 | 0 | 4 |
| Wang HM 2009 | 0 | 0 | 0 | 0 | 0 | 1 | 1 | 1 | 0 | 0 | 3 |
| Li XL 2015 | 0 | 0 | 1 | 0 | 0 | 1 | 0 | 1 | 1 | 0 | 4 |
| Li YY 2010 | 0 | 0 | 0 | 0 | 0 | 1 | 1 | 1 | 0 | 0 | 3 |
| Sai FD 2012 | 1 | 0 | 0 | 0 | 0 | 1 | 1 | 1 | 0 | 0 | 4 |
| Liu WD 2012 | 0 | 0 | 0 | 0 | 0 | 1 | 1 | 1 | 0 | 0 | 3 |
