# Supplementary material for: Comparative efficacy and safety of Chinese herbal injections combined with the FOLFOX regimen for treating gastric cancer in China: a network meta-analysis
Source: Oncotarget. 2017 Aug 18;8(40):68873–89. doi: 10.18632/oncotarget.20320 (PMC5620304; doi:10.18632/oncotarget.20320)
Supplement: Supplementary file 4 [file oncotarget-08-68873-s004.doc]

**Supplementary Table 3:** S**earch strategy**

**1. Search strategy of TCM injections**

| Name of TCM injections | English search terms | Chinese search terms |
| --- | --- | --- |
| Aidi injection | Aidi | Aidi injection OR Aidi injecta OR Aidi Ye |
| Chansu injection | Chansu OR Toad venom | Jiasu OR Shijunqing ORChandukang OR Chansu injecta OR Chansu injection |
| Fufangkushen injection | Compound matrine OR Fufangkushen OR Compound Kushen | Yanshu injecta OR Yanshu OR Fufangkushen injecta OR Fufangkushen injection OR Fufangkushen |
| Huachansu injection | Huachansu OR Cinobufacini | Huachansu injecta OR Huachansu injection OR Huachansu |
| Kanglaite injection | Kanglaite | ZCE-3 intravenous emulsion OR Coix seed extracts OR Injection of Coix seed oil OR Coixenolide OR Kanglaite injecta OR Kanglaite injection |
| Renshenduotang injection | Renshenduotang OR Ginseng polysacchride | Anerxin OR Oukanglai OR Aidean OR Changchunboao OR Baifuxin OR Renshenduotang injecta OR Renshenduotang injection OR Ginseng polysaccharide injection |
| Tongkening injection | Tongkening | Tongkening injecta OR Tongkening injection OR Tongkening |
| Wutou injection | Wutou | Taiai injecta OR Wutou injecta OR Wutou injection |
| Xiaoaiping injection | Xiaoaiping OR Marsdenia Tenacissima | Marsdenia Tenacissima extracts OR Marsdenia Tenacissima injecta OR Xiaoaiping injecta OR Xiaoaiping injection OR Xiaoaiping |
| Yadanziyouru injection | Yadanziyouru OR Javanica oil emulsion | Antikang injectaOR Yadanziyouru injecta OR Yadanziyouru injection OR Javanica oil emulsion |
| Zhulingduotang injection | Zhulingduotang OR Polyporus | Zhulingduotang injecta OR Zhulingduotang injection OR Polyporus injection |
| Delisheng injection | Delisheng | Delisheng injecta OR Delisheng injection OR Delisheng |
| Shenqifuzheng injection | Shenqifuzheng | Shenqifuzheng injecta OR Shenqifuzheng injection OR Shenqifuzheng |
| Yuanqinzhitong injection | Yuanqinzhitong | Yuanqinzhitong injecta OR Yuanqinzhitong injection OR Yuanqinzhitong |
| Huangqi duotang injection | Huangqi duotang OR Astragalus polysaccharides | Astragalus polysaccharides injection OR Huangqi duotang injecta OR Huangqi duotang injection |
| Kangai injection | Kangai | Kangai Ye OR Kangai injecta OR Kangai injection |
| Shenfu injection | Shenfu | Shenfu injecta OR Shenfu injection OR Shenfu |
| Huangqi injection | Huangqi OR Astragalus | Huangqi injecta OR Huangqi injection OR Astragalus injection |
| Xiangguduotang injection | Xiangguduotang OR Lentinan | Linengti OR Tiandixin OR Xiangguduotang injecta OR Xiangguduotang injection OR Lentinan injection |
| Shenmai injection | Shenmai | Shenmai injecta OR Shenmai injection OR Shenmai |
| Lanxiangxi injection | Lanxiangxi OR Elemene | Lanxiangxi injecta OR Lanxiangxi injecta OR β- elemene injecta OR Elemene liposome injecta OR Elemene injection |
| Banmaosuanna and Vitamin B6 injection | Sodium Cantharidinate OR Banmaosuanna | Disodium cantharidinate and vitamin B6 injection OR Banmaosuanna injecta OR Sodium Cantharidinate |
| Taipanduotai injection | Placental polypeptides | Taipanduotai injection OR Placental polypeptides injection OR Placental polypeptides |

**2. Search strategy of** **Pubmed**

#1 Stomach Neoplasms[MeSH Terms]

#2 Stomach Neoplasm[Title/Abstract]

#3 Gastric Neoplasms[Title/Abstract]

#4 Gastric Neoplasm[Title/Abstract]

#5 Stomach Cancer*[Title/Abstract]

#6 Stomach Tumor*[Title/Abstract]

#7 Gastric Cancer*[Title/Abstract]

#8 Gastric Tumor*[Title/Abstract]

#9 Gastric Carcinoma[Title/Abstract]

#10 Stomach Carcinoma[Title/Abstract]

#11 #1 OR #2 OR #3 OR #4 OR #5 OR #6 OR #7 OR #8 OR #9 OR #20

#12 Aidi[Title/Abstract]

#13 Chansu[Title/Abstract]

#14 Toad venom[Title/Abstract]

#15 Compound matrine[Title/Abstract]

#16 Fufangkushen[Title/Abstract]

#17 Compound Kushen[Title/Abstract]

#18 Huachansu[Title/Abstract]

#19 Cinobufacini[Title/Abstract]

#20 Kanglaite[Title/Abstract]

#21 Renshenduotang[Title/Abstract]

#22Ginseng polysacchride[Title/Abstract]

#23Tongkening[Title/Abstract]

#24 Wutou[Title/Abstract]

#25 Xiaoaiping[Title/Abstract]

#26 Marsdenia Tenacissima[Title/Abstract]

#27 Yadanziyouru[Title/Abstract]

#28 Javanica oil emulsion[Title/Abstract]

#29 Zhulingduotang[Title/Abstract]

#30 Polyporus[Title/Abstract]

#31 Delisheng[Title/Abstract]

#32 Shenqifuzheng[Title/Abstract]

#33 Yuanqinzhitong[Title/Abstract]

#34 Huangqiduotang[Title/Abstract]

#35 Astragalus polysaccharides[Title/Abstract]

#36 Kangai[Title/Abstract]

#37 Shenfu[Title/Abstract]

#38 Huangqi[Title/Abstract]

#39 Astragalus[Title/Abstract]

#40 Xiangguduotang[Title/Abstract]

#41 Lentinan[Title/Abstract]

#42 Shenmai[Title/Abstract]

#43 Lanxiangxi[Title/Abstract]

#44 Elemene[Title/Abstract]

#45 Sodium Cantharidinate [Title/Abstract]

#46 Banmaosuanna[Title/Abstract]

#47 Placental polypeptides[Title/Abstract]

#48 #12 OR #13 OR #14 OR #15 OR #16 OR #17 OR #18 OR #19 OR #20 OR #21 OR #22 OR #23 OR #24 OR #25 OR #26 OR #27 OR #28 OR #29 OR #30 OR #31 OR #32 OR #33 OR #34 OR #35 OR #36 OR #37 OR #38 OR #39 OR #40 OR #41 OR #42 OR #43 OR #44 OR #45 OR #46 OR #47

#49 randomized controlled trial[Publication Type]

#50 controlled clinical trial[Publication Type]

#51 randomized[Title/Abstract]

#52 placebo[Title/Abstract]

#53 randomly[Title/Abstract]

#54 trial[Title/Abstract]

#55 groups[Title/Abstract]

#56 "drug therapy" [Subheading]

#57 #44 OR #45 OR #46 OR #47 OR #48 OR #49 OR #50 OR #51

#58 animals[MeSH Terms]

#59 humans[MeSH Terms]

#60 #58 NOT #59

#61 #57 NOT #60

#62 #11 AND #48AND #61

**3. Search strategy of** **Embase**

#1 random*

#2 placebo*

#3 doubl* adj blind*

#4 singl* adj blind*

#5 assign*

#6 allocat*

#7 “double-blind procedure”/exp

#8 ”randomized controlled trial”/exp

#9”single-blind procedure”/exp

#10 #1 or #2 or #3 or #4 or #5 or #6 or #7 or #8 or #9

#11 ''Stomach tumor'/exp

#12 Stomach Neoplasms

#13 Gastric Neoplasms

#14 Gastric Neoplasm

#15 Stomach Cancer

#16 Stomach Tumor*

#17 Gastric Cancer*

#18 Gastric Tumor*

#19 Gastric Carcinoma

#20 Stomach Carcinoma

#21 Stomach Cancers

#22 #11 OR #12 OR #13 OR #14 OR #15 OR #16 OR #17 OR #18 OR #19 OR #20 OR #21

#23 aidi

#24 chansu

#25 toad AND venom

#26 compound AND matrine

#27 fufangkushen

#28 huachansu

#29 cinobufacini

#30 kanglaite

#31 renshenduotang

#32 ginseng AND polysacchride

#33 tongkening

#34 wutou

#35 xiaoaiping

#36 marsdenia AND tenacissima

#37 yadanziyouru

#38 javanica AND oil AND emulsion

#39 zhulingduotang

#40 polyporus

#41 delisheng

#42 shenqifuzheng

#43 yuanqinzhitong

#44 huangqi AND duotang

#45 astragalus AND polysaccharides

#46 kangai

#47 shenfu

#48 huangqi

#49 astragalus

#50 xiangguduotang

#51 lentinan

#52 shenmai

#53 lanxiangxi

#54 elemene

#55 Sodium Cantharidinate

#56 Banmaosuanna

#57 Placental polypeptides

#58 #23 OR #24 OR #25 OR #26 OR #27 OR #28 OR #29 OR #30 OR #31 OR #32 OR #33 OR #34 OR #35 OR #36 OR #37 OR #38 OR #39 OR #40 OR #41 OR #42 OR #43 OR #44 OR #45 OR #46 OR #47 OR #48 OR #49 OR #50 OR #51 OR #52 OR #53 OR #54 OR #55 OR #56 OR #57

#59 #10 AND #19 AND #58
