# Supplementary material for: Comparative efficacy and safety of Chinese herbal injections combined with the FOLFOX regimen for treating gastric cancer in China: a network meta-analysis
Source: Oncotarget. 2017 Aug 18;8(40):68873–89. doi: 10.18632/oncotarget.20320 (PMC5620304; doi:10.18632/oncotarget.20320)
Supplement: Supplementary file 5 [file oncotarget-08-68873-s005.doc]

**Supplementary Table 4. More details about the product information of CHIs**

| CHI name | Raw materials | Labeled efficacy | Indications |
| --- | --- | --- | --- |
| Compound kushen injection | *Sophorae Flavescentis*  *Radix, Smilacis Glabrae*  *Rhizoma* | Clearing away hot, removing dampness, cooling blood, removing toxin, resolving hard mass, and relieving pain | Cancer pain, bleeding |
| Shenqifuzheng injection | *Astragali Radix, Ligustri Lucidi Fructus* | Tonifying Qi and nourishing Yin | Fatigue, lack of strength, vertigo caused by asthenia of pulmonosplenic Qi; auxiliary treatment of the above symptoms for lung cancer and gastric cancer |
| Aidi injection | *Mylabris, Ginseng Radix Et Rhizoma, Astragali Radix, Acanthopanacis Senticosi Radix Et Rhizoma Seu Caulis* | Clearing away hot, removing toxin, resolving stagnation and dispersing masses | Primary liver cancer, lung cancer, rectal cancer, malignant lymphoma, gynecological malignant tumors, etc. |
| Kangai injection | *Astragali Radix, Ginseng Radix Et Rhizoma, Sophorae Flavescentis Radix* | Tonifying Qi and strengthening  body resistance | Primary liver cancer, lung cancer, rectal cancer, malignant lymphoma, gynecological malignant tumors; neutropenia and thrombocytopenia; chronic hepatitis B from various causes. |
| Huachansu injection | *Bufonis Corium* | Removing toxin, detumescence, and relieving pain | Middle and advanced cancer, chronic hepatitis B etc.. |
| Kanglaite injection | *Jobstears Seed Oil* | Tonifying Qi, nourishing Yin, and resolving mass | Unfavorable operation of Qi and yin deficiency, spleen deficiency and dampness retention with primary non-small cell lung cancer and primary liver cancer; has anti cachexia and analgesic effects in patients with advanced cancer |
| Javanica oil emulsion injection | *Bruceae Fructus* | Anticarcinogen | Lung cancer, brain metastasis of lung cancer and digestive system neoplasms |
| Xiaoaiping injection | *Marsdenia tenacissima Caul* | Clearing away heat and toxic material, resolving phlegmand softening hard masses | Esophageal cancer, gastric cancer, lung cancer, liver cancer; combination with radiotherapy and chemotherapy as adjuvant therapy |
| Delisheng injection | *Radix Ginseng, Astragalus, venenum bufonis, cantharides* | Supplementing Qi and strengthening the body resistance, eliminating inflammation and dissipating stasis | Syndromes of Qi deficiency stasis on middle-late stage primary liver cancer, abdominal mass in right rib, pain of fixed location, abdominal distension, fatigue. |
| Elemene injection | β-, γ-, δ-elemene mixture | / | Combined with routine regimen of radiotherapy and chemotherapy to enhance the curative effect and reduce the toxic and side effects for treating lung cancer, liver cancer, esophageal cancer, nasopharyngeal cancer, brain tumor, bone metastasis cancer and other malignant tumors; interventional therapy, intracavitary chemotherapy and treatment of carcinomatous hydrothorax and ascites; as first-line treatment for malignant pleural effusion, ascites, brain tumors, respiratory tract and digestive tract tumors; as second-line therapy for gynecologic cancer, breast cancer, skin cancer, bone metastasis, lymphoma, leukemia, etc.. |
| Shenmai injection | *Radix Ginseng Rubra, Radix Ophiopogonis* | Tonifying Qi and preventing exhaustion, nourishing Yin and generating body fluid, activating pulse | Shock, coronary heart disease, viral myocarditis, chronic pulmonary heart disease and neutropenia with deficiency of Qi and Yin; improve the immune function of patients with tumors, combined with chemotherapy to enhance the curative effect and reduce the toxic and side effects |
| Disodium cantharidinate and vitamin B6 injection | Disodium cantharidinate and vitamin B6 | Anticarcinogen | Advanced primary liver cancer and advanced lung cancer |
| Chansu injection | *Venenum bufonis* | Heat-clearing and detoxicating | Acute and chronic suppurative infection; as adjuvant drug for malignant tumors |
| Lentinan injection | Lentinan | Immunomodulator | Adjuvant therapy for malignant tumors |
| Ginseng polysaccharide injection | Ginseng polysaccharide | Immunomodulator | Alleviate the side effects of radiotherapy and chemotherapy, adjuvant therapy for tumors; improve the immune function, acute and chronic hepatitis and liver injuries, chronic infections, diabetes and immunological disease |
| Shenfu injection | *Radix Ginseng, Radix Aconiti Carmichaeli* | Reviving Yang for resuscitation, tonifying Qi and preventing exhaustion | Desertion syndrome caused by excessive Yang Qi desertion (infectious, hemorrhagic and fluid loss shock); deficiency of Yang or Qi with palpitation, cough, stomachache, diarrhea, rheumatism, etc. |
| Astragalus polysaccharides injection | Astragalus polysaccharides | Supplementing Qi and tonifying deficiency | Fatigue, spontaneous sweating, shortness of breath, anorexia of Qi deficiency syndrome; patients with aleucocytosis, low quality of life, and immunocompromise after chemotherapy |
| Astragalus injection | *Astragali Radix* | Tonifying Qi and strengthening  body resistance, pulseinvigorating and heartnourishing, fortifying spleen  and disinhibiting dampness | Insufficiency of the heart-qi and blood stasis syndrome of viral myocarditis, cardiac insufficiency; hepatitis with spleen deficiency and dampness syndrome |
| Tongkening injection | *Equisetum hiemale, Selaginella tamariscina* | Heat-clearing and detoxifying, expelling stagnation and relaxing vein | Adjuvant therapy for malignant tumor-induced pain |
| Wutou injection | *Radix Aconiti, Radix Aconiti Kusnezoffii* | tranquilizing and relieving pain | Stomach cancer, liver cancer and other advanced cancers-induced pain |
| Polyporus polysaccharide injection | Grifola polysaccharide | Immunomodulator | Combined with antineoplastic drugs, it can enhance the curative effect and a decrease side reactions |
| Yuanqinzhitong injection | *Corydalis ambigua Ch., Radix Aconiti Praeparata, Fraxinus bungeana, Radices Stephaniae Tetrandrae, Chelidonium majus L.* | Promoting Qi to activate blood, activating meridians to stop pain | Moderate cancerous pain caused by Qi stagnation and blood stasis |
| Placental polypeptides injection | Placental peptide | / | Reduced or dysregulated cellular immunity-induced disease, postoperative healing, viral infection-induced disease, leucopenia caused by various reasons |
